# Barriers and enablers to primary health care center access for older people in Lebanon: A qualitative inquiry

**DOI:** 10.1371/journal.pone.0335073

**Published:** 2025-10-23

**Authors:** Saydeh Dableh, Kate Frazer, Mathilde Azar, Randa Hamadeh, Thilo Kroll

**Affiliations:** 1 School of Nursing, Midwifery, and Health Systems, University College Dublin, Dublin, Ireland; 2 Nursing Program, University of Balamand, Beirut, Lebanon; 3 Primary health care department, Ministry of Public Health, Beirut, Lebanon; University of Nigeria - Enugu Campus, NIGERIA

## Abstract

**Introduction:**

Older people in low- and middle-income countries face significant challenges when accessing primary care services. In Lebanon, most older people (75%) living with at least one chronic disease previously accessed private health services for care. However, the economic crisis substantially increased their reliance on primary healthcare centers (PHCCs), while factors shaping access to public services were unknown. This study explores the barriers and enablers influencing access to PHCCs’ services.

**Methods:**

This descriptive qualitative study involved 57 people including older adults (aged 60–92 years), informal caregivers, and service providers, recruited using maximum variation sampling. Data were collected through seven focus groups and fifteen interviews. The Framework Method was adopted for thematic analysis. The Patient-centered Access to Healthcare Framework facilitated mapping of barriers and enablers across five access opportunities.

**Results:**

Findings are presented under five themes: 1) *perception of healthcare needs*, enabled by acute symptoms, free services, literacy, and familial support but hindered by lack of information on services; 2) *healthcare seeking*, supported by respectful providers, familial support, available quality services, and positive leadership, but constrained by providers’ attitudes, poor service organization, limited finances, and negative perceptions; 3) *healthcare reaching*, enabled by proximity of PHCCs and home care, but limited by transport issues, mobility restrictions, staff and resource shortages, and service delivery challenges; 4) *healthcare utilization*, facilitated by low fees and economic recession, but hindered by lack of funds and financial resources; and 5) *healthcare consequences,* facilitated through positive relationships, literacy, and personal abilities, but constrained by cognitive and sensory limitations, poor relationships, and lack of care continuity, coordination, comprehensiveness, and patient-centeredness.

**Conclusions:**

This study highlights the challenges for older people, indicating factors to be strengthened and barriers requiring action at the PHCC and multi-sectoral levels. Ensuring adequate funding, information, and health coverage is primordial to improve older people’s access to PHCCs.

## Introduction

Enhancing older people’s access to Primary Healthcare (PHC) services is crucial for providing comprehensive and person-centered care without financial hardship [1]. Strengthening PHC systems is globally recommended to adapt to demographic and epidemiological transitions [2] and fulfill international commitments toward achieving Universal Health Coverage (UHC) [3]. It also improves health outcomes, upholds equity, and fosters health system efficiency [2]. Despite these benefits, access to PHC services remains an understudied issue in many Low- and Middle-Income Countries (LMICs) [4], where health systems often fail to achieve UHC [5,6]. This is particularly concerning because most older people (80%), defined as individuals aged sixty and above [7], reside in these countries [8] that face significant resource constraints and health inequities [9].

In LMICs, the use of PHC services among older people varies significantly. It depends on socioeconomic determinants [10], geographic factors [11], and the specific context of healthcare delivery [12]. Moreover, older people’s access to PHC services is particularly limited during pandemics, due to mental health challenges and lack of digital literacy [13]. Two recent systematic reviews summarized the factors that facilitate and constrain older people’s access to PHC services in LMICs, identifying influences related to both health system attributes and older people’s characteristics [12,14]. Factors enabling access to PHC include family support, proximity to PHC settings, positive provider attitudes, enhanced mobility, and free services. Conversely, barriers include negative attitudes from providers, a shortage of health professionals, a lack of integrated and person-centered care, and long travel distances. While Kwaitana et al. (2024) noted limited research in this area, Dableh et al. (2024) highlighted the need to explore PHC access at the country level, considering contextual factors and stakeholders’ perspectives.

Understanding the factors that influence older people’s access to PHC services can help develop equitable policies [15] and people-centered restructuring of PHC to meet their unique needs [16] and perspectives [17]. Timely access to health services supports well-being, quality of life, and improved health outcomes. [16,18].

In Lebanon, 11% of the population is aged 65 years and older and uses over 60% of healthcare resources [19]. Most older people (75%) live with at least one chronic disease, and nearly 50% have no health insurance [19]. The healthcare system in Lebanon is mainly privatized, fragmented, and focused on disease treatment [20]. The Ministry of Public Health (MOPH) currently allocates only 5% of its budget to primary and preventive care [21]. About 30 years ago, the MOPH established a national PHC network as part of health service reforms. This network of 237 Primary Health Care Centers (PHCCs) was formed by contracting with existing dispensaries across Lebanon [20,21]. The MOPH did not provide any financial support to the PHCCs. Instead, funding depends on many Non-Governmental Organizations (NGOs) and local charities [20,21].

Additional strains on the PHC system and delivery of services within PHCC include the Syrian crisis and workforce shortages [nurses and physicians] [20–22]. The PHCCs’ ability to provide equitable services has been strained from the beginning, and access for the Lebanese population was limited until the impact of the 2019 economic crisis. Reduced income and inability to pay for private healthcare forced many to access PHCCs, which were an affordable alternative [23,24]. The use of PHCC services increased by 100% in 2021 compared to 2019 [25], but the experiences of increased demand on the fragmented, underfunded services were not explored. This study aims to identify the barriers and enablers influencing older people’s access to PHCCs in one Lebanese community and to inform ongoing service development and policy.

## Materials and methods

### Conceptual framework of the study

**Fig 1:**
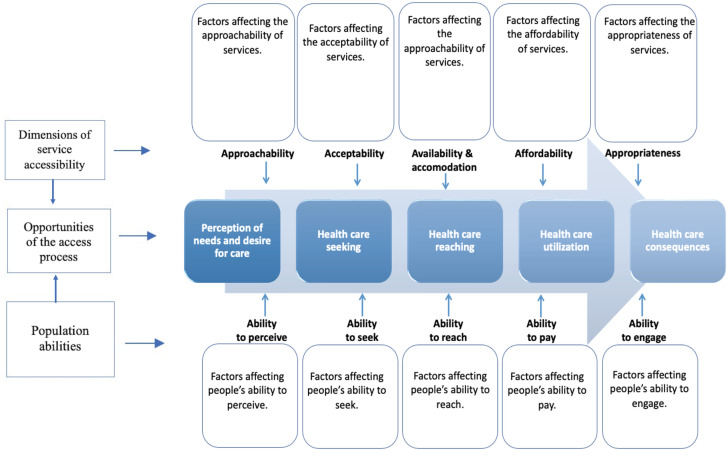
Illustration of the Patient-Centered Access to Health Care Framework- adapted from [26] for illustrative purposes.

The Patient-Centered Access to Health Care Framework guided the data collection and analysis. In 2013, Levesque et al. summarized various definitions of healthcare access through a comprehensive review of available literature. Their access framework established a five-point model including the opportunity to perceive the need for care, to seek healthcare services, to reach healthcare resources, to use these services, and to attain healthcare consequences. The process is operationalized through the interaction of five service dimensions from the supply side, each mirrored by a corresponding population ability from the demand side [26] (Fig 1). This framework was selected for this study as it 1) allows a comprehensive conceptual assessment of access to PHC services that extends beyond service utilization 2) considers both demand and supply aspects, and 3) has been successfully used by researchers to analyze healthcare access across different contexts, target groups, and care levels [4].

### Study setting

Lebanon is a middle-eastern country with a population estimated at around six million people [27]. About 11% are aged 65 and older; the highest percentage in the Arab world [19]. Since 2019, the country has experienced one of the worst economic recessions of the twenty-first century, and its reclassification from a higher middle-income country to a lower middle-income country. Between 2019 and 2021, its GPA dropped by 36% [28].

We conducted this study in Koura, a suburban district in the North Lebanon governorate, which is a peripheral area lacking institutions that provide healthcare services for older people (OP). There are 84,600 people living in the Koura region with 10.9% of them over the age of 65 years [29]. The majority (78.5%) of people living in Koura describe their economic status as average or lower [29]. We selected two PHCCs from five located in Koura, representing different levels of funding support, staff mix composition, and beneficiaries’ size and cultures. The first PHCC is funded by the municipality and served 6887 people in 2023. The health care team is formed of 10 full-time workers, including two nurses, along with part-time providers covering 12 specialties. The second PHCC is funded by external agencies and served 20000 people in 2023. There are 12 full-time health care workers, including 5 nurses, along with part-time providers covering 14 specialties.

### Study design

This is a qualitative descriptive study guided by the pragmatism paradigm, which emphasizes practical solutions to real-world problems [30,31]. Drawing on the literature [16], we chose a design that enables a deep understanding of older people’s experiences with accessing PHC. To ensure reporting rigor, we followed the Standards for Reporting Qualitative Research (SRQR) [32].

### Participants and recruitment

This study involved four types of stakeholders: 1) OP who use services delivered through PHCCs, labelled as “users” or “UP” in this study 2) OP who do not use services provided through PHCCs, designated as “non-users” or “NUP” 3) family members of users “FMU” and non-users “FMNU” and 4) primary care service providers designated as “SP”. Including users and non-users along with service providers allows capturing different perspectives on access barriers and enablers from both the supply and demand sides. We employed a maximum variation sampling strategy to recruit participants differing based on PHCC usage, age, gender, and socio-economic status. PHCC focal people recruited service users, providers, and their family members, while municipal focal people recruited non-users and their family members. SD met with these focal people to explain the study and recruitment process. After being informed, interested individuals contacted SD to participate. Table 1 presents the number and selection criteria for each participant category. It also reflects the anticipated minimum number of participants pre-set to generate rich data that captures the complexity of the ‘access’ phenomenon. This estimate considers several criteria [33,34], including the research question, diversity and inclusion, available resources, and existing literature on acceptable sample sizes. The final sample size was determined based on a combination of factors: a) the achievement of sufficient depth in the data, reflected in well-developed themes and the absence of new insights [35]; b) the authors’ interpretive judgment regarding the adequacy of the data to meet the analytical goals and address the research question [36]; and c) a pragmatic assessment of the value of collecting additional data considering the available time, personnel, and financial resources [33].

**Table 1:** Participants and recruitment plan.

Participants	Recruitment	Initial plan for recruitment and data collection	Actual number of participants and data collection methods	Inclusion criteria	Exclusion criteria	Selection Method
Older people using PHCCs	Through 2 PHCCs in Koura	12-16 ^(a)^ in 2 FGDs 5-8 Individual interviews ^(b)^	1 FGD in the first PHCC (n = 6) 1 FGD in the second PHCC (n = 6) 8 Individual interviews Total: 20 participants	Lebanese ^(c)^ males and females Aged 60 years old and over ^(d)^ Having different socio-economic statuses Users of diverse PHC services, including medical consultation, for at least twice during the past year Being able to provide consent for themselves	Older people living in institutions ^(e)^	Purposive Sampling
Older people NOT using PHCCs	Through two municipalities where PHCCs are located	6-8 participants in 1 FGD 5 Individual interviews	1 FGD (n = 6) 6 Individual interviews Total: 12 participants	Same as for users, unless for use of PHCCs Have decided not to seek services delivered through PHCCs	Older people living in institutions	
Service providers	Through 2 PHCCs in Koura	5-6 in one FGD	1 FGD in the first PHCC (n = 6) 1 FGD in the second PHCC (n = 4) ^(f)^ Total: 10 participants	Having at least one year of professional experience within PHCCs Serving older people		
Family members	Through two PHCCs and municipalities where PHCCs are located	8-10 participants in 1 FGD	1 FGD for family members of users (n = 6) 1 FGD for family members of non-users (n = 8) ^(g)^ 1 interview Total: 15 participants	Close relatives Older people are dependent on them		

(a) The number of people participating in FGD is decided based on similar studies included in the scoping review [37]. A smaller number of participants in each group can facilitate discussions especially if they need more time to express themselves effectively.

(b) This number will ensure diversity in terms of the presence or absence of sensory/motor/cognitive limitations. Authors can advise on increasing the number of participants if deemed necessary.

(c) Only Lebanese older people will be involved as access patterns differ substantially between locals and refugees [23].

(d) In Lebanon, older people are defined as people aged over 65 [19], however for this study, we adopted the UN definition of older people [9], those aged 60 years old or over.

(e) Older people living in institutions will be excluded as they receive PHC within their residential institutions.

(f) One FGD took place in each of the two PHCCs, as it was difficult to join staff operating in both PHCCs together in one FGD.

(g) Family members of users and non-users were split into two different FGDs because one non-user older women highlighted during her interview an attitude of her children toward seeking care from PHCC. Therefore, we decided to separate family members so they can report their viewpoints freely.

### Data collection

SD and MA developed topic guides in English after discussions with TK and KF, translated them into Lebanese Arabic, and tailored them into participant categories using Levesque et al.’s framework (S1 File). Informed by the Lebanese context and existing evidence [16], the guides covered selected service dimensions and population abilities: approachability, acceptability, affordability, and appropriateness along with their corresponding abilities to perceive, seek, pay, and engage with care. SD conducted 7 Focus group Discussions (FGDs) and 15 individual interviews between November 2023 and January 2024. FGDs (90 minutes) included all participant categories and were held at PHCCs or an accessible venue, while interviews (20–45 minutes) targeted older people with motor and sensory limitations and were conducted at their homes. Data, including socio-demographic details (Table 2) and views on access barriers and enablers, were collected in Arabic and audio-recorded with consent.

**Table 2:** Older people’s characteristics.

Older people (n = 32)	(n)	(%)
**Use of PHCCs**		
Users-yes	20	62.5
Non-users-no	12	37.5
**Age**		
60-70	10	31
71-79	17	53
≥ 80	5	16
**Gender**		
Male	15	47
Female	17	53
**Economic status** ^**(1)**^		
Low	17	53
Medium	14	44
High	1	3
**Occupation**		
Currently working	2	6
Not working	30	94
**Education**		
None	1	3
Primary-secondary	24	75
University	7	22
**Health insurance** ^**(2)**^		
Public	15	47
Private	3	9
None	15	47
**Chronic disease (self-reported)**		
None	3	9
1-2	18	56
≥ 3	11	35
**Functional limitations (self-reported)** ^**(3)**^		
None	21	66
Cognitive limitation	0	0
Sensory limitation	6	19
Motor limitation	7	22
**Living arrangement**		
Alone	5	16
With a family	27	84
**Reach to the PHCC**		
Travel independently	21	66
Need assistance	9	28
Cannot travel/require homecare	2	6
**Travel time to reach the nearest PHCC**		
Up to 5 minutes	10	31
6–20 minutes	20	62.5
21–30 minutes	2	6.5
**Transportation means**		
Walking	4	12.5
Own car	23	72
Private taxi	5	15.5

(1)Economic status as perceived by participants.

(2)One participant can have a public and a private insurance.

(3)One participant can have more than one type of limitations.

### Data analysis

SD manually transcribed the audio recordings verbatim, read the transcripts, and recorded initial impressions, allowing a deep immersion in the data. She analyzed the data in Arabic, with 15% of transcripts translated into English for independent coding (SD, KF, and TK) and peer debriefing. To ensure quality translation, an independent bilingual professional at University College Dublin back-translated a random selection of four pages and then discussed the results with SD to compare them with the original text. Discussion with TK, KF and SD confirmed the translations. Using the Framework Method [38], SD analyzed data concurrently with its collection, applying an initial analytical framework based on Levesque et al.‘s framework and relevant literature [16] (rationale and process are included in S2 File detailing the application of the Framework Method). A combined inductive and deductive coding approach allowed the identification of both expected and emerging views from participants’ accounts. SD, KF, and TK independently coded early translated transcripts and discussed interpretations. All FGDs and interviews were analyzed as one dataset, and the framework was iteratively refined throughout the analysis process (S3 File). Coded data were charted into a framework matrix (example in S2 File) allowing comparisons across participants and the development of descriptive accounts [38,39]. Quotes were translated and presented in both languages (S4 File).

### Rigor and trustworthiness

Strategies adopted to meet rigor criteria [40] included prolonged engagement with the data; presenting the findings to a subset of participants; peer debriefing; development of thick contextual descriptions; independent coding of 15% of transcripts; and maintaining an audit trail. A reflexive journal using the “what” model [41] was maintained. The research team were experienced in both qualitative methods and understanding of the context of PHC in Lebanon. The investigator’s professional experience in PHC and caring for older people, combined with contextual knowledge, influenced the choice of the research topic along with key methodological decisions, including recruitment of participants through independent gatekeepers.

### Ethical consideration

This study was approved by the UCD Health Research Ethics Committee (LS-CO-23–185- Dableh-Kroll, November 23, 2023), the IRB of the University of Balamand (IRB-REC/O/023-23l0923, July 2023), and the Primary Health Care Department at the Lebanese MOPH (468PHC, August 1, 2023). Participants provided written consent after receiving information about the study including details on their voluntary participation and the opportunity to withdraw. Participants were informed about the secure storage of data and the maintenance of their confidentiality with results to be presented as group summaries to protect identities.

## Results

### Participants’ characteristics

**Fig 2:**
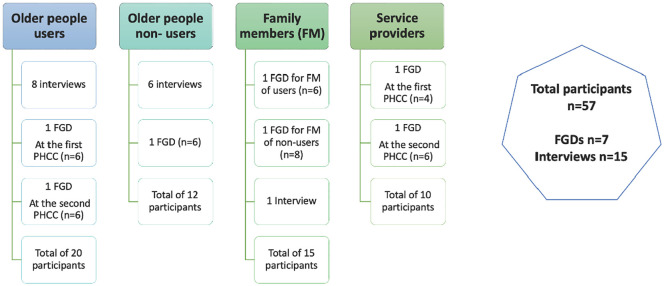
Participants and data collection.

Fifty-seven participants took part in FGDs and interviews as summarized in Fig 2. The characteristics of OP, family members, and service providers are detailed through Tables 2– respectively. OP were aged between 60 and 92 years, with 53% pertaining to the 71–79 age group. They represented different gender, socio-economic statuses, and health needs. Most of them live within families which reflects the Lebanese context. Family members caring for an older relative were aged between 30 and 65, with the majority ranging from 51 to 65 years old. They represented different socio-economic statuses, with a majority being females who were caring for OP, although not necessarily living with them. Service providers included administrators and care providers with varying years of experiences with the majority being fully committed to PHCCs.

**Table 3:** Family members’ characteristics.

Family members (n = 15)	(n)	(%)
**Age**		
30-40	3	20
41-50	4	27
51-65	8	53
**Gender**		
Male	5	33
Female	10	67
**Economic status** ^**(1)**^		
Low	7	47
Medium	8	53
High	0	0
**Occupation**		
None	8	54
Private sector	5	33
Public sector	2	13
**Education**		
Primary	1	7
Secondary	6	40
University	8	53
**Health insurance** ^**(2)**^		
None	4	27
Public	8	53
Private	3	20
**Marital status**		
Married	10	67
Single	5	33
**Degree of kinship**		
Son	5	33
Daughter	9	60
Sister	1	7
**Lives with older people**		
Yes	4	27
No	11	73

_(1) Economic status as perceived by participants._

_(2) One participant can have a public and a private insurance._

**Table 4:** Service providers’ characteristics.

PHC providers (n = 10)	(n)	(%)
**Role at the PHCC**		
Physician	2	20
Nurse/nurse assistant	4	40
Non-medical staff	1	10
Administrative/reception	3	30
**Years of experience at PHCCs**		
Up to 10 years	5	50
More than 10 years	5	50
**Location of practice**		
Exclusive PHCCs	8	80
PHCCs & private clinics	2	20

### Access barriers and enablers

Drawing on Levesque et al.’s framework, findings are presented under five themes representing the five access opportunities: perception of needs and desire for care, healthcare seeking; healthcare reaching; healthcare utilization; and healthcare consequences. Under each theme, subthemes represent the corresponding service dimension and population ability. S4–S6 Files provide supporting details, including respectively quotes in Arabic translated to English, code classification into categories, subthemes, and themes, along with a mapped summary of access barriers and enablers.

#### Theme 1: Perception of needs and desire for care.

##### 1.1. Approachability

**Information on available services**

While service providers and a few users referred to the increased number of beneficiaries to say that OP know about PHCCs, all other participants agreed that a remarkable lack of knowledge exists regarding the whole concept of PHC and the PHCCs. This includes mainly the location of centers, available services, eligibility and registration mechanism, funding sources, and service organization. Participants used dispensaries and PHCCs interchangeably without recognizing the difference between them.

*“This is the first time we hear about this program, about primary care”* (NUP, male, 71-79 age group).

While participants highlighted the lack of awareness and advertising, a few users noted that information is available online but remains inaccessible to illiterate individuals.

**Source of information**

Information about PHCCs is disseminated through formal and informal channels; however, participants agreed that word of mouth was the primary source of information about PHCCs and that people with lived experiences and PHCCs staff members themselves play an active role in communicating information. Disseminating information through social media platforms, flyers and banners, community meetings with key people, and outreach activities were examples noted by participants as formal channels to share information.

##### 1.2. Ability to perceive

**Socio-demographic determinants**

*Education and health literacy*

While some participants stated that education does not interfere with the ability to perceive the need for care, other participants mentioned that educated people read more and become aware of the symptom significance, disease complications, importance of early detection and preventive measures. They differentiated between education and health literacy, stating that both enable this ability, especially when coupled with previous lived experience.

*“My sister knows everything, she’s not educated but she has experience”* (FMU, female, 51-65 age group)

*Age*

While a few participants believe that age enables the care need perception, all other participants agreed that OP in Lebanon get care only when in need and at advanced stages.

*“Like cars, until they turn off”* (NUP, male, ≥80 age group)

*Living arrangement*

While some participants argued that living alone enables the need perception because of the higher prevalence of negative health conditions, others stated that family members note the occurrence of symptoms and discuss with the OP the need for care.

“*Family members recognize the symptoms of the disease and encourage the older person to seek medical care”* (FMU, female, 41-50 age group)

*Socioeconomic status*

Several participants noted that the perceived need for care increases with people’s ability to afford it.

*“Wealthier people are more likely to desire care than those who cannot afford it”* (UP, Male, 71-79 age group).

*“They fear going to the hospital and discover the need for further care; some people cannot afford the hospitalization cost, that’s why they prefer skipping explorations, they postpone investigations”* (UP, female, 60-70 age group)

**Health determinants**

*Health needs*

All participants agreed that health need is the main driver for OP to start the healthcare access journey. Need encompasses mental, functional, and physiologic issues and refers to any perceived symptoms, malfunction, weakness, pain, anxiety, and chronic disease management. Despite their awareness about preventive services, OP present late to services and seek care often at advanced stages.

*“99% of Lebanese people seek care at a final stage”* (NUP, male, 60-70 age group)

*Emotional status*

Emotional status was described as stress and fear. Participants mentioned that poor life conditions and stressors lead to sadness and dissatisfaction, thus increasing health problems, which in turn increase the need for health services, thereby enhancing the perception of need among OP. Health providers noted that some people with emotional distress seek counseling at PHCCs.

*“I feel that sometimes they need to talk, they like to come and talk, we listen to them”* (SP, up to 10 years of experience)

Participants stated that fear can have contrasting effects on OP’s perception of healthcare: it may prompt those concerned about their health to seek care, but it can also discourage those who associate illness and medical treatment with death, viewing care as the beginning of a degenerative decline.

*“He would say I don’t want to do the checkup, I prefer not to know”* (FMNU, female, 41-50 age group)

*Cognitive abilities*

The impact of cognitive abilities was noted especially for those with reduced ability and had an effect on their perception of need for care, their ability to verbalize needs, often leading to high endurance, consisting of an increased ability to live with comorbid symptoms.

*“Cognitive limitations impair the ability to recognize and express the need for care”* (NUP, male, ≥80 age group)

**Service-related determinants**

*Free services*

Older users and non-users reported that perceiving the need for care increases with the provision of free services, such as preventive screening campaigns.

*“I got motivated to seek care when I saw advertisements for free blood test campaigns” (UP, male, 71-79 age group)*

#### Theme 2: Healthcare seeking.

##### 2.1. Acceptability

**Provider-related determinants**

*Staff behavior and attitude*

According to users’ experiences, staff attitude and behaviors vary across centers; however, all participants agreed that this factor is key to service acceptability, shaping the overall image of PHCCs. Treating people humanely, welcoming them warmly and respectfully, listening actively with compassion, and delivering organized services enhance service acceptability. However, poor communication, disorganized services, rude and careless behaviors, and unprofessional conduct like favoritism and politicizing services are unacceptable. Yelling and talking with superiority are provided as examples of aggressive communication while not respecting the queue order was mentioned as an example of poorly organized services. Overcrowding was seen as a trigger for staff frustration, which can negatively impact client interactions.

*“When I go to the hospital outpatient clinic, they welcome me warmly. When I went to the medical center, she made me feel indebted for the favor of providing services”* (FMNU, male, 30-40 age group).

*Provider’s characteristics*

Participants mentioned that close and familial relationships with providers facilitate access. While some noted that Muslim women may prefer female providers, most agreed that provider gender does not impact acceptability. OP value being able to choose their providers and prefer those who are experienced, specialized, and demonstrate humane and ethical behavior. Some service providers reported that certain physicians undermine PHCCs by portraying them as low quality to preserve patients at their private clinics and maximize their financial benefits.

*“When you know that the attending physician is competent and treats you with respect you will definitely go to the primary care center”* (UP, female, age group 71-79)

**Service-related determinants**

*Service organization*

Participants from all categories reported that long waiting times (up to 4 hours) in overcrowded PHCCs are a main barrier resulting from high demand and physicians’ delayed arrival. Overcrowding is accompanied with the need to stand and line up and attitudes that bother OP like pushing, and nagging.

*“I am an old-women, I am sick and have body aches, I cannot go there and sit for three to four hours to get examined. This is constraining our ability to get medications, and we need these medications”* (UP, female, ≥ 80 age group)

Waiting for long is particularly difficult for OP and family members who have roles and functions to fulfill.

*“My mom is an old-women, I cannot leave her alone for four to five hours while waiting to see the physician”* (FMU, female, 51-65 age group).

Participants stated that while overcrowding and waiting times are acceptable when services are well organized, favoritism and skipping the queue are not. Efficient service organization was reported as prioritizing OP based on case severity rather than age, which promotes access for older people with cognitive or motor limitations.

*“Once they see him at the wheel chairs, he doesn’t wait, even a minute”* (FMU, female, 41-50 age group)

*Availability and quality of services*

Older people expressed that high-quality services fulfilling their needs are well accepted, especially the availability of chronic medications, medical equipment, and skilled providers. However, both older people and family members highlighted that a lack of information, particularly about medication funding and the benefits of donations that are initially dedicated for refugees, reduces service acceptability.

*“Older people are encouraged when they know the provider, and know how services are funded and why they are low- cost” (UP, female, 60-70 age group)*

**Cultural and social determinants**

*Trust in governmental services*

Participants conveyed mixed views on trusting governmental services. Non-users particularly stated that public services are generally distrusted, except for PHCCs due to successful vaccination programs and international support. While complaints about staff attitude and service quality were common, participants expressed that trust in PHCCs depends on the leadership and management of each center.

*“There is a shared information that if you go to primary health care centers affiliated to the Ministry of Health, you won’t get a decent reception; Lebanese people consider that governmental or free services are below the required level”* (NUP, male, age 60-70 age group)

*Cultural aspect of the center*

Participants reported differing viewpoints on the acceptance of services delivered through PHCCs that have clear political or religious affiliations. While some users and service providers believe that PHCCs provide services to everyone without discrimination other users and non-users confirmed that they do not seek services delivered by centers that have clear religious or political affiliation, with political affiliation being the most intolerable.

*“I personally do not like to seek care from a center with political affiliation, I feel I do not accept, I do not enter it, but I do not mind if it has a different religious affiliation”* (FMNU, female, age 41-50 age group)

*Influx of Syrian displaced people*

The influx of Syrian displaced people to PHCCs induced controversial views regarding its influence on the acceptance of services. Some users supported inclusion on humanitarian and equity basis but admitted that the number of people moving into the country contributed to overcrowding and long wait times, which was noted as particularly challenging for OP. Most older participants and family members, however, expressed their frustration, feeling that Lebanese citizens were a minority among “strangers”, endure competition for services, and struggle to afford even minimal fees while perceiving that non-Lebanese people did not have to pay due to humanitarian supports. The conditions and overcrowding dissuaded some participants from accessing PHCC as they perceived they owed their access to international aid.

*“When I come to the center and I see this overcrowd and chaos, and non-Lebanese people are also seeking medications, it makes me feel that the center is not appropriate for me, I won’t come back*” (UP, male, age 71-79 age group)

Service providers emphasized that tensions were greater at the beginning of the Syrian crisis and eased over time.

*Shared experiences*

Participants from all categories declared that OP share their experiences with access to PHCCs and influence their peers’ help seeking decisions. For example, shared positive experiences with staff welcoming and respect is encouraging people to seek services delivered by one of the PHCCs. However, non-users reported that they were influenced by peers reporting negative experiences like low-quality services, lack of medication, and discontinuity of a care.

*“The center reputation motivates people to come, satisfied beneficiaries will send their peers, welcoming is being consistently great, it is not individual”* (UP, female, age 71-79 age group)

*Critical negative perception*

Participants across all groups highlighted three key perceptions that constrain OP’s access to PHCCs. First, PHCC users are labelled as for the poor or disadvantaged populations, making care-seeking feel like a loss of dignity; some family members fear being criticized for seeking care for their parents from PHCCs. Care providers reported that people who became obliged to seek PHCCs services after the economic crisis felt ashamed.

*“I know many people who think that PHCCs are for the poor, that’s why they don’t go”* (NUP, male, ≥ 80 age group)

Second, PHCC services are perceived as providing lower quality care, with less competent providers compared to private clinics.

*“A perception that the quality is low, not good, deficient. Neither physicians nor medications, nothing”* (SP, more than 10 years of experience).

Third, non-users believe that by using PHCC services, they might be taking resources away from those who are more disadvantaged and in greater need.

##### 2.2. Ability to seek

**Socio-demographic determinants**

*Socioeconomic status*

All participants noted that financial abilities strongly influence care-seeking. Limited income and lack of insurance were reported as key barriers, driving OP to delay care, ignore preventive measures, and only seek help when symptoms become severe.

*“My brother passed away because of a colon cancer; we as brothers shall do a colonoscopy every two years, I haven’t done it in the past 4 years, I can’t afford it*” (UP, male, age 60-70 age group)

However, participants clarified that limited financial abilities affect the choice of healthcare settings, making PHCCs a more viable option, especially for those who can no longer afford private care, particularly after the economic crisis. They feel obliged to endure difficult conditions, including long waiting hours and jeopardized dignity, in order to access needed services.

*“The financial factor after all is currently the main driver for people to seek care from PHCCs” (*NUP, female, 71-79 age group)

Most OP depend on their family members to pay for healthcare services. Therefore, they under-report symptoms and delay care to relieve the related financial hardship.

*“We’re living on my son’s expenses; I feel ashamed to add to his burdens, I feel anxious about this because he has a family”* (UP, female, 71-79 age group)

*Gender*

Some participants reported that women fear sickness and seek care more than men do. Men were more prone to delaying care and bearing symptoms.

*“While I bear any symptom, like most men, my wife seeks care for every simple condition”* (UP, male, 71-79 age group)

*Education and health literacy*

While most participants mentioned that education and health literacy are enablers to seeking care, a few others highlighted that educated people seek more care but not from PHCCs. A service provider noted that education does not make a difference with advanced age, for those with cognitive limitations.

*“Educated people recognize symptoms early and know that they should seek care since they are aware of complications”* (NUP, male, 60-70 age group)

**Social and cultural determinants**

*Family and social support*

Participants from all categories reported that OP who have family members or connected through a social network seek more care compared to people who live alone. Family members notice symptoms, encourage OP to seek care, and participate in choosing the care setting and the provider. Family members stated that emotional support provides the OP with motivation to get better and pursue life. Neighbors can provide instrumental support through calling and accompanying OP to care settings. Service providers highlighted that OP whose children are employees or live abroad face difficulties in seeking care and present late for care at advanced stages in the disease process.

“*They do not have someone to care for them, to accompany them, or their family members are employees, so until the case becomes advanced so one of their family members bring them, if they have family members”.* (SP, up to 10 years of experience)

Service providers confirmed that the presence of a family member at the care setting also enhances the quality of care by providing accurate data and assisting the OP in understanding instructions and procuring needed resources for different treatment strategies. OP who attend alone can hide their prescriptions or conceal their illness to avoid bothering their family members.

*Roles and functions*

Older people stated that those who still have financial responsibilities tend to seek more care, as they need to maintain good health. However, housewives and OP who care for other family members like grandchildren do not have capacity to wait for long hours to access services at PHCCs.

*“They maintain their health not just for themselves, but because their families depend on them”* (FMU, female, 41-50 age group)

*Religion*

According to OP and family members, religious beliefs influence the health seeking decisions especially regarding the use of centers with clear religious affiliation. Moreover, some people may delay care and rely on God for symptoms healing.

*“We’re still rooted in the cultural background no matter what”* (NUP, male, ≥80 age group)

*Personal beliefs and attitudes*

As described by participants, these factors refer to four different mindsets among OP; first, non-users who can afford other options, believe that they should not abuse services delivered by PHCCs thus take advantage over poor people, especially that available resources are limited. This contrasts with the beliefs of OP who like to take advantage of any free available services irrespective of their financial abilities. Second, some OP bypass settings and cost to adhere to the same provider, constraining the use of services delivered by PHCCs.

*“I prefer going directly to the hospital, instead of trying new settings”* (FMNU, female, age 41-50 age group).

Finally, individuals who distrust public services or suppose that low-cost care reflects poor quality, doubting also the providers’ competence, are less likely to use PHCC services. Family members confirmed that seeking care from private settings is a form of respect for OP, especially that seeking care from PHCCs can induce social judgement and gossip.

*“They feel ashamed to use cheap or free services”* (UP, female, 71-79 age group)

**Health determinants**

*Emotional status*

According to participants’ accounts, emotional status implies fear and sadness. While several participants reported fear and concern about their health as an enabler to seek care, others reported fear as a barrier; they described OP’s fear of medical examination, fear of disease, fear of getting contaminated at care settings especially during pandemics, fear of being humiliated by staff attitude, but most commonly fear of uncovering a disease that requires unaffordable care. Sadness is another barrier to seeking care; providers mentioned that people who are depressed are less likely to seek care especially at early stages. Family members confirmed that successful ageing and loving life are enablers to seeking care.

*“You feel that most older people in Lebanon are so sad, they are depressed and feeling heavy, they do not feel comfortable asking for anything or seeking help, they would like to live shorter”* (NUP, female, 71-79 age group)

*Physical and mental abilities*

Participants highlighted that cognitive limitations increase dependency and limit the ability to seek care. Physical dependency was used to describe motor limitations and inability to call and drive.

*“It is difficult for me to make phone calls; I do not know how to do.* (UP, female, 71–79 age group)

*Health needs*

Participants from all categories gave different connotations to the “need” concept. It refers to the case acuity that determines the choice of the care setting; PHCCs are chosen for low-acuity cases and hospitals for more serious ones. The need for medication pushes OP to seek services delivered anywhere including PHCCs. Having specific needs may constrain OP from waiting for long hours at PHCCs.

*“When a person needs medications, he goes anywhere just to get it”* (NUP, female, 71-79 age group)

**Environmental determinants**

*Travel distance and transportation*

A long distance to reach the PHCC combined with unavailability of transportation and high transportation cost were reported as barriers to seeking care from PHCCs.

*“It is very easy for us, we come here by walking”* (UP, female, age 71-79 age group)

#### Theme 3: Healthcare reaching.

This study did not aim to assess this opportunity of the access process; however, participants reported during discussions challenges that pertain to the availability and accommodation dimensions and to older people’s ability to reach services, as described through Levesque et al.’s framework.

##### 3.1. Availability and accommodation

**Service-related determinants**

*Availability of professionals*

The participants reported many barriers related to the availability of the professionals. The presence of physicians at PHCCs is limited to a few hours per week based on a schedule which can result in delayed care and overcrowding. Staff shortage in some PHCCs and the lack of presence of a general practitioner during all opening hours, especially in the afternoon, is considered as a barrier to timely care. In contrast, the presence of skillful physicians from all specialties is recognized as an enabler to PHCCs access. Service providers highlighted that work conditions at PHCCs are not appealing or enough to sustain, hence, they cannot be fully dedicated to work at PHCCs. Service providers reported that centers are well staffed but staff lack specific geriatric training.

*“We attended many trainings last year, but none related to older* *people”* (SP, up to 10 years of experience).

*Availability of medications and equipment*

Service users and providers agreed that the lack of chronic medication is a main barrier. Service providers added that while the MOPH supplies medications for acute conditions every three months, the Youth Men’s Christian Association (YMCA), an INGO, is the only supplier of chronic medications and their program present several limitations: 1) The number of subscribers is restricted with difficulty to add beneficiaries and cope with the increased demand; 2) the YMCA provides a predetermined list of medications lacking advanced ones and others to treat musculoskeletal and neurological diseases like Alzheimer and epilepsy; 3) due to dwindled resources, the YMCA is not supplying enough medications to cover all subscribers’ needs. This results in dissatisfaction and treatment discontinuation. Beneficiaries are only taking medications that are retrieved at the PHCC as they cannot afford the high cost of medications provided through pharmacies. Thus, they skip doses or make amendments without referring to their physicians, against the international guidelines for treatment of certain conditions like diabetes and hypertension.

*“Most people are getting two medications out of three, three out of five. They do not buy the remaining, they cannot afford. They are taking only the medications that are provided here, not the full prescription”* (SP, up to 10 years of experience).

Moreover, service users confirmed that some physicians are referring PHCCs beneficiaries to their private clinics as the required equipment to complete the diagnosis in certain cases is lacking. Providers highlighted also the lack of assistive devices that help OP with physical limitation to reach into the center.

*Scope of services*

Service providers reported that PHCCs do not offer specific geriatric services beyond chronic diseases management. One of the two PHCCs offers homecare which is highly appreciated and recognized as an access enabler when performed with high quality. However, the number of allowed homecare visits per month is limited, enabling access mainly for people with cognitive and motor limitations.

*“A physician and nurse visit me monthly and bring my medications, otherwise I wouldn’t be able to get care, I cannot go to the center in my case!”* (UP, female, 71-79 age group)

Some assistive programs that are “relieving for households” (SP, up to 10 years of experience) of people with motor limitations are discontinued due to fund shortage. Non-users described services offered by PHCCs as inappropriate for older people as they do not include some needed services. Users notably reported the lack of specialized care, medications, diagnostic tests, screening tests, daily blood testing, health education, and vaccines for older people.

*Service organization*

Participants reported several access barriers and enablers related to service organization.

Enablers included the possibility to call and make appointments; organized waiting arrangements; comfortable waiting rooms equipped with Televisions; short waiting time; limited number of patients per hour of examination with fair payment to physicians; availability of personnel to assist the walk in of older people with disability; reminder calls to pick-up medications; outreach activities to promote preventive tests; computerized records; and prioritizing OP. However, barriers included restricted opening hours that are unsuitable for family members of OP; overcrowding; long waiting times (over two hours); too many scheduled appointments per hour and overcrowding; inappropriate conservation and packaging of medications; unfair payment for physicians; and disorganized waiting arrangements with favoritism.

*“Sometimes when you go to the center and you find this overcrowd, 20 patients to be examined in one hour, you would leave, you cannot get good results amidst this overcrowd”* (UP, female, ≥80 age group)

Service providers mentioned that opening hours, number of patients per hour of examination, and physician payment vary across centers. Physicians are paid either per patient or per visit. Most of them are not interested in extending their schedule and presence at PHCCs as they are not well paid; they rely on their private clinics to sustain. Moreover, decreased funds per month is resulting in reduced opening hour as attempts to limit expenses and maintain budget.

*“Paying the physician per patient may affect the quality of care as physicians could speed up the examination to see more patients per hour, especially when need is high*” (SP, more than 10 years of experience).

**Environmental determinants**

*Travel and transportation*

The participants mentioned that Koura district is not adequately connected through common transportation. Moreover, transportation cost surged after the economic crisis increasing also private and taxi fees which constitutes an access barrier especially for OP who do not drive.

*“Public transportation is not available, ordering taxi is expensive, and it is hard to walk to the PHCC especially in extreme weather”* (UP, female, 60-70 age group)

Considering high transportation costs, travel distance mainly determines participants’ choice of care settings. Proximity of PHCCs is an access enabler for users, with a 30-minute travel considered as far.

*Built environment*

Service providers highlighted that PHCCs are selected by the MOPH based on criteria ensuring their accessibility. They are either located on the ground floor of buildings or equipped with functional lifts over 24 hours, with the availability of ramps for wheelchair access, which are considered access enablers. Some OP reported constrained access to clinics located on higher floors when lifts are lacking. They also refrained from undertaking their preventive tests when lifts were not available during the economic crisis due to a lack of electricity supply.

*“Diabetic patients need help, indeed, to take the stairs and reach the ophthalmologic clinic at the second floor”* (FMU, female, 51-65 age group)

##### 3.2. Ability to reach

**Socio-demographic determinants**

*Age*

Participants agreed that advanced age limits some OP’s physical abilities thus, constrains their ability to access PHCCs.

*“Driving my father out of home is a real struggle”* (FMNU, male, 30-40 age group)

*Dwelling areas*

Participants reported that living in urban areas characterized with short distances and transportation connections and social support enables OP’s ability to reach PHCCs. Living in overpopulated areas can constrain access to PHCCs because of overcrowding.

*“My patients in Tripoli [Lebanese city] easily reach to the care setting, but centers there are overcrowded”* (SP, more than 10 years of experience)

**Health determinants**

*Functional and instrumental abilities*

Participants stated that limited mobility, physical disability, the inability to drive, and the lack of assistive devices like wheel chairs constrain OP’s access to PHCCs.

“*I get out to his car to perform blood testing as wheel chairs are lacking at our center”* (SP, up to 10 years of experience)

#### Theme 4: Healthcare utilization.

##### 4.1. Affordability

**Economic determinants**

*Availability of geriatric funds*

Service providers clarified that PHCCs within the national network rely on either municipal or INGO funding, each facing distinct challenges. Municipality-funded PHCCs endured severe financial shortages during the economic crisis. They lack specialized geriatric programs and depend on medical consultation fees to sustain operations. INGO-funded PHCCs previously offered programs like homecare for OP with disabilities. However, dwindling external funds, diverted to global emergencies like wars and conflicts, resulted in discontinuation of these services.

*“We used to get large funds, now it’s almost nothing”* (SP, up to 10 years of experience).

According to service providers, the ability of PHCCs to provide services is decreasing while the demand is increasing especially after the economic crisis. One of the centers stopped covering the diagnostic tests fees to be able to provide basic services for a larger number of beneficiaries. As for chronic medications, they are supplied for all PHCCs, exclusively through an INGO, the YMCA. After the economic crisis the number of subscribers to the chronic medication program surged drastically while the supply decreased due to limited funds.

*“The need in this region is exceeding the support we are getting”* (SP, up to 10 years of experience)

*Transportation cost*

All participants mentioned that the transportation cost surged after the economic crisis and OP prefer using the nearest healthcare setting or omitting care especially when it comes to preventive tests, even free ones, to save the transportation cost.

*“Public transportation is not available, ordering taxi is expensive, and it is hard to walk to the PHCC especially in extreme weather”* (UP, female, 60-70 age group)

**Service-related determinants**

*Service fees*

Participants presented controversial views whether low-cost constitute an enabling or constraining factor to access PHCCs services. Many participants believe that low-cost services encourage people to use services provided by PHCCs especially that they cannot afford the cost of services provided by the private sector after the economic crisis.

“*People are not able anymore to afford services provided at private clinics, neither the cost of medical consultations not the cost of diagnostic tests*” (FMU, female, 51-65 age group)

Remaining participants stated that low-cost is a constraining factor as it is correlated to low-quality services with inferences even made on physicians’ capabilities. However, they agree that most people who refused to use PHCCs services prior to the economic crisis, are seeking now those services as they are not able anymore to afford healthcare expenses at the private settings. Some participants affirmed that low-cost does not affect the acceptability of medications but it affects the quality of examination and diagnostic tests.

“*I personally fear low-cost services, I see them as deficient, if I am able, I won’t choose them”* (FMNU, female, 41-50 age group)

*Contribution fees*

One additional enabler reported by service providers is applying specific considerations regarding the payment of people with disabilities and people with financial limitations. Beneficiaries are exempted from paying fees based on rapid or other needed extensive assessment.

*“Sometimes it is easy to identify beneficiaries who lack financial means. Those are fully exempt of paying fees”* (SP, up to 10 years of experience)

##### 4.2. Ability to pay

**Socio-demographic determinants**

*Socioeconomic status*

According to participants’ accounts, the general financial status of OP determines their health seeking behaviors. Financial abilities result from the income, savings, nature of the previous work, and familial support.

They confirmed that people who are financially stable seek care from private settings rather than waiting for hours at the PHCCs to get services.

*“Well-off people won’t go wait for three to four hours at the primary care center to get the service. They seek care from the hospital outpatient clinic and pay as long as they can afford the cost”* (UP, female, 60-70 age group)

However, OP with limited financial abilities or financial dependency delay or omit care and seek services at PHCCs. They added that OP who are still able to work and earn income seek more care compared to people who lost their working abilities. Moreover, the situation of OP who used to get pensions changed after the economic crisis coupled with currency devaluation.

*“The older person bears as much as he can these days” (UP, female, 71-79 age group)*

*Insurance and pension plans*

Participants agreed that having insurance enables the access to health care in general; however, insured people seek care from private settings rather than PHCCs. Access also depends on the type of insurance especially after the economic crisis that disrupted the functionality of all public insurances.

Service providers highlighted that private insurances are expensive and unaffordable after the economic crisis. Insurance agreements vary but in general do not make significant difference at the PHCCs level as they cover hospitalization and diagnostic tests but does not cover medical consultation and medications. Older people stated that national schemes covering employees within the private and public sectors (like the National Social Security Fund- NSSF and the COOPerative of public sector employees-COOP for private and public sectors respectively) are based on out-of-pocket payments then re-imbursements. Hence, some OP lack financial resources to make the payment and then wait for reimbursement.

*“I am insured with the National Social Security Fund, but cannot get care if I do not have money”* (NUP, female, 60-70 age group)

OP and family members reported that such schemes got completely dysfunctional during the economic crisis due to currency devaluation. Beneficiaries who used to be covered up to 100%, were obliged to seek care from PHCCs, in the absence of other alternatives, or endure financial hardship to cover care expenses at private settings. OP are anxious about health complications that may require hospitalizations.

*“The older person lives with an obsessive idea, a constant mental distress: what do I do if any health problem happens to me?!”* (UP, male, 71-79 age group)

Some users declared that other schemes exist for military corps but beneficiaries can get care exclusively at their specific centers which requires sometimes long-distance travels. In view of the increased transportation cost, those beneficiaries are recently using PHCCs. Now, more than ever, OP complain about the lack of governmental support and wish old-age insurance and pension plans were available to relieve them from this mental burden due to their increased financial dependency and jeopardized dignity.

*“The lack of old-age insurance is essential. The government is absent!”* (NUP, male, 71-79 age group)

*Familial and social support*

OP nowadays rely on the financial support provided by family members living in Lebanon but mostly those living abroad. The Lebanese diaspora is also financially supporting the Lebanese community in different ways. Participants reported that the presence of such support, enables access to PHC.

“*If one’s is lacking external support, he cannot live and sustain, at all. This is my personal experience”* (NUP, male, 71-79 age group)

**Economic determinants**

*The economic crisis*

OP and family members described the effects of the unprecedented economic crisis compounded with currency devaluation and bank system failure, on their help seeking behaviors. The ability to pay crumpled with the collapsed savings at banks and the value of retreated employees’ pensions drastically dropped (2000 $ prior to crisis value around 30$ in 2024). Moreover, OP who used to be insured, pay at the private sector at a rate of 100000 Lebanese pounds for each US dollar, and get reimbursed through banks at the rate of 1500. They feel humiliated because of the whole process.

*“I took the invoice and waited for more than an hour and a half in front of the bank, like a beggar, to get reimbursed. Two hours in front of the ban to collect pennies from the invoice”* (UP, female, 60-70 age group)

They added that the inflation and high living expenses are changing the families’ and OP’s priorities. The deteriorating financial status disabled OP’s access to healthcare in general but catalyzed the acceptance and use of services delivered through PHCCs.

*“Today’s income is not enough even to get a medical examination. One has multiple bills; we are resetting our priorities because our savings collapsed at banks”* (FMNU, male, 51-65 age group)

#### Theme 5: Healthcare consequences.

##### 5.1. Appropriateness

**Service-related determinants**

*Geriatric clinical examination*

Participants reported that the average of the clinical examination duration at PHCCs is 10 minutes (5 minutes the lowest and 15 the highest), compared to 30 minutes at private clinics (20 minutes being the lowest and 45 minutes the highest). The clinical examination consists of two parts: 1) patient preparation completed by nurses including measurement of vital signs height and weight, and data collection; 2) clinical examination completed by physicians including case discussion, diagnosis based on the chief complaint, and prescription.

While non-users perceived the examination quality at PHCCs as poor compared to private clinics, users’ views regarding the quality of the clinical examination varied within the same center and across different centers. Satisfied users reported that the duration is “appropriate” and physicians discuss plentifully with no rush. They found that nurses are qualified and contribute to the examination through performing preparation, doing some tests like ECG and glucose test.

*“As in private clinics, a nurse prepares you and accompany you to the physician who dedicates appropriate time” (FMU, female, 30-40 age group)*

Unsatisfied participants reported that the clinical examination is shallow, focused on the chief complaint, lacking comprehensive geriatric assessment, with inconsistent completion of patient preparation. Table 5 illustrates complaints about the clinical examination with supportive arguments and quotes. Participants believe that the restricted examination time resulting from the high demand and unfair payment is affecting the quality of examination.

**Table 5:** Complaints about the quality of clinical examination.

Complaints	Arguments	Supportive quotes
Superficial clinical examination	Consists mainly of talking with frequent interruptive phone calls. The physical examination is performed only if needed. Some physicians underestimate the patients’ chief complaints and consider them as normal part of the ageing process. Users and providers agreed that physicians explain strictly what is necessary while writing, without maintaining eye contact. The basic care consisting of diagnosing and prescribing medication is delivered; however, the extensive case discussion and answering all patients’ questions, that is “part of the case management” (physician, above 20 years of experience) is not possible at PHCCs. Homecare quality and duration varied across centers; while some participants reported that it lasted 30 minutes and the professionals showed a careful and professional attitude, others mentioned that it was rushed and that providers did neither care for the patient’s chief complaint nor provided follow-up information.	*“Consisting only of questions, just talking with no attempts to measure your pulse or put a stethoscope to check your lungs and heart” (UP, male, 71-79 age group)* *“When they assign me thirty or forty patients per hour, I cannot even look at their eyes” (SP, more than 10 years of experience)* *“No, no, no, no, he didn’t touch me. They said we will do now a blood test then I will examine you later on. They did not even tell me how and when to get the results” (UP, male,* ≥*80 age group)*
Inconsistent application of patient preparation	While service providers confirmed that patient preparation is integral part of the patient examination, users of one center stated that it was not applied consistently especially when the center is overcrowded. Physicians highly appreciate the support provided by nurses but confirmed that patients do not count patient preparation as part of the examination as it is performed by nurses and not physicians.	*“Sometimes they measure my blood pressure when I ask for this, but not every time before seeing the physician” (UP, female, 71-79 age group)*
Body system-focused Clinical examination	At PHCCs and even in private clinics, the majority of specialized providers ask exclusive questions related to the system they care for. For example, cardiologists deal merely with cardiovascular problems. At PHCCs the explanation is precise and concise and does not cover indications and side effects of prescribed medications most of the time. Physicians do not have time to review all file details. Therefore, family members feel responsible to memorize the medication list and summarize the case for the physician on behalf of OP.	*“They focus only on your chief complaint” (NUP, male, age 71-79 age group)* *“I need to be a half doctor to understand the case and explain it to the physician” (FMU, female, 40-50 age group)*
Lack of comprehensive geriatric assessment	The geriatric assessment is neither comprehensive and structured, nor systematically applied in all centers. At one of the centers, service providers apply twice a year, tools provided by the Ministry of Public Health to assess the mobility, psychological health, mental health, and sensory problems among all people aged above 18. However, providers at the other center recalled such tools available through the informatic system but never applied. Even when applied, the assessment tools do not cover all geriatric problems. Urinary incontinence, nutrition and hydration, and reproductive health remain under-screened. Physicians, irrespective of the setting type, do not apply any specific age-related protocol during their clinical examination. Memory and mental problems are evaluated through general discussion without using geriatric assessment tools. Holistic care, as reported by providers, consists of checking all medications, dealing with all complaints, and transferring patients to other specialists when necessary.	*“Honestly, I have never come across a doctor who elaborates on these matters. He always focuses on your disease and prescribes treatment, but he doesn’t go into detail such if you have a familial support, your life, how you feel, how you deal psychologically with events and your illness. It rarely happens. We lack this culture in Lebanon”. (UP, male, 71-79 age group)* “*Memory problems do not fall under his specialty” (NUP, male, 71-79 age group)*

*Care coordination*

According to participants, family medicine does not serve as a gate to the healthcare system in Lebanon. For every set of symptoms, the patient chooses a different specialized physician who stays focused within his scope of specialty. Family members highlighted the absence of individual computerized record accessible for providers across settings, which makes care coordination difficult and depending on individual initiatives. Family members try to fill in this gap by memorizing all details to provide comprehensive information about the older person’s case when needed.

*“We need to memorize all details because we don’t have a comprehensive medical record”* (FMNU, female, 41-50 age group)

Service providers confirmed that a computerized record for each patient exists at PHCCs; however, physicians refuse to fill in these files as they consider it as additional work that they are not keen to do especially considering unfair payment. Nurses are replacing physicians to complete the files. Care providers mentioned that included information is available internally to the interdisciplinary team members, though, the restricted examination time is a main barrier for this coordination channel. External referrals are only done through the system to get approvals on diagnostic tests rather than for coordination purposes.

*“The issue is that physicians do not want to do additional work. They may say we are earning one or two dollars for each patient, so we won’t manage data on top of this, they do not accept”* (SP, up to 10 years of experience)

*Care continuity*

Users reported that they are usually examined by the same physician at the PHCC. As for medications, the YMCA is not providing sufficient quantities of all medications to cover all subscribers’ needs which leads to treatment discontinuation. This was a main concern reported by the majority of participants. Similarly, service providers confirmed that different programs funded by external agencies were discontinued because of ceased funds.

*“We have a project funded by IMC (NGO) to support people who are aged … this was a great project, but it is being stopped now” (*SP, up to 10 years of experience)

**Provider-related determinants**

*Care comprehensiveness*

Participants agreed that care providers, at all settings, provide mainly therapeutic care without emphasizing preventive care, with the exception of some family medicine specialists at their private clinics. For example, information about available vaccines and age-related screenings for different types of cancers are overlooked. However, the participants added that some specialized physicians ask about some related screenings; the urologist may suggest a prostate cancer screening and the gynecologist a mammogram. Such tests could be also requested for diagnosis rather than preventive purposes, especially when the beneficiary presents specific symptoms. Health education activities are seldom offered at centers and rarely attended by beneficiaries, especially after the rising transportation cost.

*“Every specialized physician will delve into his field. For example, a gynecologist might discuss mammograms and such things. But if he is a generalist, he won’t ask the lady if she has done a test for cervical cancer”.* (FMU, female, 51-65 age group)

All participants agreed that from the demand side, Lebanese OP seek care only when in need and at advanced stages; this could be either related to lack of awareness, to fear of discovering age-related malfunction, because of considering themselves too aged, or most commonly because of their short financial resources, especially after the economic crisis. Some participants linked it to cultural issues.

“*You no longer think about yourself except about what is necessary. For example, I waited to the point that I was not able to see with my eyes anymore, that I went to the physician”* (UP, female, 71-79 age group)

*Client-provider relationship*

Participants approved that a trustful client-provider relationship affects the quality of the examination and improves health outcomes.

“*Getting on well with the care provider is important to tell him everything. If you don’t feel comfortable, you won’t say anything”* (NUP, male, 60-70 age group).

Service providers mentioned that OP are privileged at PHCCs considering their age. However, many important factors affect their relationship with their providers including the user’s attitude and behaviors, the payment system, the overcrowding, in addition to the professionals’ ethical conduct, consciousness, and training on communication with OP.

Satisfied users mentioned relationship boosters including explanation in plain language, quality care irrespective of the setting type, consideration of OP’s concerns, questioning, active listening, counseling, follow-up calls, OP prioritization, cultural competence, and users’ awareness about the case.

*“I am noticing that you need to get some awareness about your case, to be able to discuss with the physician, so you force him to provide time for discussion*” (FMNU, male, 41-50 age group)

Non-satisfied users highlighted barriers to respectful relationship with providers including negative communication like yelling, getting angry of people who ask, not maintaining eye contact, expressing rude verbal statements, and using technical terms to explain. Other factors include the lack of explanation, careless attitude, lack of time to interact and set a pleasant environment, paternalistic approach, disinterest regarding the social and financial aspects of beneficiaries, materialistic approach, and under-estimation of OP’s complaints.

*“He taps on your shoulder and that’s it. He says: you’re in good health mam, you have nothing. You should be thankful you’ve got to this age; I hope we age like you and stay that strong!”* (UP, female, 71-79 age group)

##### 5.2. Ability to engage

**Socio-demographic determinants**

*Education and health literacy*

While some participants stated that education does not interfere with the OP’s ability to engage, others reported that educated people engage better in their care plan discussions.

*“I feel shy to ask because I am not educated”* (UP, female, 71-79 age group).

However, all participants agreed that irrespective of education, health literacy enables OP to engage, to understand directives, and to promote the quality of the assessment.

*Trust*

Participants reported that some OP over trust their physicians. They consider them as knowledgeable and accountable for their prescriptions, thus they manifest a submissive attitude by neither asking nor arguing.

*“Do I know as much as the physician?! I do whatever he says”* (UP, female, 71-79 age group)

*Self-efficacy and interest*

Participants reported that OP who have enhanced self-efficacy and curiosity dare to ask more than others who do not like to discuss. They mentioned that the interest to know more about the health condition, the medications, and treatment alternatives that allow savings also motivates OP to ask. Interest increases when OP are in pain or present a serious or acute condition.

*“Participation depends on the patient’s personality and condition”* (UP, female, age 71-70)

**Health determinants**

*Cognitive and sensory abilities*

Participants agreed that preserved cognitive and sensory abilities enable OP to ask questions and engage in discussing their care plans. However, OP with cognitive limitations might not recall their list of medications, understand directives, or provide accurate information. Service providers affirmed that showing up unaccompanied at PHCCs can lead to fatal medical errors. Sensory limitations are also considered by participants as access barriers.

*“Some people have hearing limitations, they would say, even if I ask, I won’t be able to hear the answer, so they do not ask” (SP, up to 10 years of experience)*

*Emotional status*

According to participants, OP who fear sickness ask more to gain knowledge about their case and avoid complications, however, OP who fear physicians and medical examinations engage less and get confused and submissive during the clinical examination. Service providers highlighted that sad and depressed OP are less likely to engage with care discussions. They do not have the ability to discuss and argue, they lack focus, and get easily confused. The same case applies to OP who are hopeless and forced by the family members to get care.

*“Some patients are hopeless to the extent of not caring for their treatment and health” (SP, over 10 years of experience)*

**Provider-related determinants**

*Perceptions around providers’ conduct*

Participants agreed that providers play a significant role in enabling OP to engage in discussing their care plans. Warm welcoming, caring for their concerns, asking about them, and following up on their issues are provided as helpful providers’ conduct fostering a positive relationship and facilitating OP’s engagement.

“*If the patient feels comfortable with the physician, he will ask, but when he feels that the physician is providing strict and short answers and not tackling further issues, he feels the need to stop engaging, he won’t continue” (NUP, female, 60-70 age group).*

*Perceptions around providers’ communication skills*

The users noted communication barriers constraining OP’s engagement including the provider getting rude over OP who argue, not explaining or saying what is strictly necessary, serious facial expressions, and talking while writing.

*“He got mad, he gave me the prescription paper and pen and told me: you prescribe!”* (UP, male, 71-79 age group)

The users mentioned that positive communication encouraging them to interact includes asking questions, listening actively, maintaining eye contact, drawing to explain, explaining while using plain language, being approachable through phone calls.

*“Nothing matters, but only if he [the physician] listens to me” (*UP, female, 71-79 age group)

**Social and cultural determinants**

*Role of family members*

Participants reported that OP rely on their family members who play a significant role in the decision making related to their care plan. They summarize to the provider complicated cases, seek information, and discuss choices. They feel responsible and worried thus, they ask questions to get reassured. OP enjoy family member’s interference perceived as a form of care, especially when the provider keeps on addressing both.

*“The family member needs to know the case to explain to the physician”* (FMNU, female, 41-50 age group)

Alternative source of information

Participants mentioned that they rely on search engines like Google to get needed information and make informed decisions when physicians do not explain.

*“I ask my son, he searches the web for medication side effects”* (UP, female, 71-79 age group)

**Service-related determinants**

*Examination time and fees*

Some OP believe that arguing and getting information is part of their right as they pay for the examination at private clinics. However, the clinical examination tight duration and nominal fees at PHCCs limit this ability.

*“It gets problematic when the physician is overwhelmed” (FMNU, Male, 51-65 age group)*

## Discussion

**Fig 3:**
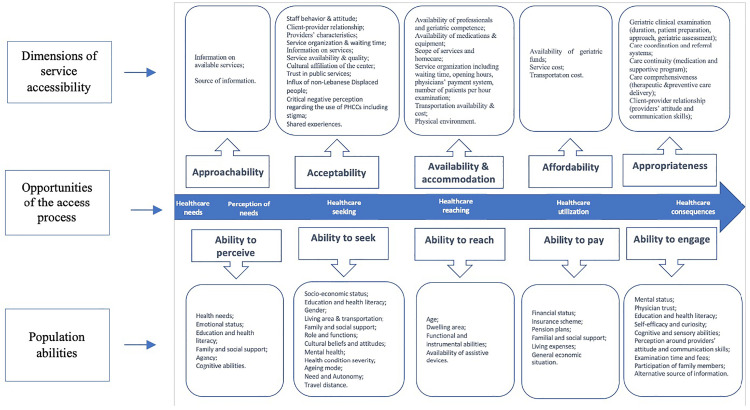
The adapted Patient-Centered Access to Health Care Framework.

This study aimed to identify the enablers and barriers to OP’s access to PHCCs in Lebanon, as perceived by OP, family members, and service providers. Results show that factors affecting access to PHCCs pertain to all five access opportunities outlined in the Patient-centered Access to Health Care Framework (Fig 3).

The perception of needs and the desire for care are affected by a significant information gap about PHCCs. Most participants were unfamiliar with the concept of PHC and the Lebanese PHC network, including the locations of centers, eligibility criteria, available services, funding sources, and differences between dispensaries and PHCCs. Service providers hesitated to invest in informational campaigns and outreach activities because they feared attracting more people while their funds were dwindling. PHCCs that rely on local government funding have been experiencing difficulties after the economic crisis. Others relying on external agencies also have limited funding as new emergencies occur worldwide. The evidence shows that health marketing improves access to care [42–44]. However, precluding such campaigns to limit demand can lead to unmet healthcare needs for populations, exacerbate health disparities, miss preventive opportunities, and reduce healthcare system efficiency [45]. Instead of suppressing demand, a more effective and comprehensive approach would involve targeted outreach [42], optimizing existing resources, designing sustainable financing schemes [46], and addressing the social determinants of health that contribute to poor health outcomes [1]. From the demand side, the desire for care among most OP is mainly triggered by disease occurrence, particularly at an advanced stage. They might ignore preventive care and seek PHCCs’ services related to their chronic disease management, notably the provision of medications, medical consultations, and blood tests. Family support, stressful events resulting in poor health outcomes, fear of disease, and the availability of free health services are also reported as additional triggers for care. Enhancing OP’s health literacy appears crucial to counter their beliefs that enduring symptoms show strength and that preventive care is the initial step toward degenerative diseases.

At the healthcare-seeking level, many factors affect service acceptability and the OP’s ability to seek care. The professional, caring, and respectful attitude of staff members appears to be a key component for accepting services provided by PHCCs, as also reported in available literature from LMICs [12,14]. The positive communication skills and ethical conduct of healthcare providers are essential to build trustful client-provider relationships, improve the quality of the examination, and engage OP and family members in their health decision-making. This corroborates findings from Westerling et al. (2022), who also reported that older people feel safe when treated in calm environments by welcoming and caring professionals [47]. The findings also reflect that the acceptability of services is linked to their availability, quality, and organization, which depend on local leadership and management. Two major issues negatively impacted the acceptability of services. First, the stigma around PHCC users reflects a shared perception that PHCCs primarily serve the most disadvantaged people by providing low-cost services, which are perceived as low-quality. Second, there was a lack of acceptance toward Syrian displaced individuals seeking healthcare services at PHCCs, which were funded by NGOs. The participants in the study identified that the massive influx of displaced people led to a decrease in the use of PHCCs by the Lebanese, who already had limited resources. Identifying displaced individuals as the cause of the problem could be mistaken for racism or xenophobia. Lebanese OP who lacked access to healthcare themselves reflected in this study the pain of seeing “foreigners” benefiting from a full package of services made exclusively available for them for free, at the same centers where these OP failed to get the same needed services and struggled to pay even the nominal fees. However, this discrepancy points out a lack of information about refugee support programs funded by NGOs and channeled directly to PHCCs. It also reflects the inconsistent management of the health system, which simultaneously directed people toward PHCCs while withholding information that these facilities were already overcrowded and had long waiting times. To restore the trust of Lebanese citizens in PHCCs, the MOPH implemented a restoration program in 2016, aiming to provide a package of primary care services to the poorest Lebanese families [21]. Targeted families were mostly satisfied; however, the low-use trend persisted among the non-targeted citizens until the economic crisis [25] in 2019 forced more Lebanese citizens to rely on PHCC services. The system left them feeling offended at PHCCs, judged as “non-suitable” for most of them, and had to either seek care from private settings or seek services delivered through PHCCs despite all the challenges, with a reported sense of “humiliation”. The increased use of PHCCs by Lebanese people has highlighted the challenges of limited services, which are not directly funded by the government but rather through third parties, including municipalities, NGOs, and charities. Disorganized services and negative provider attitudes leading to shared negative experiences seem to exacerbate trust issues in PHCCs’ services, especially those operated by political parties. From the demand side, participants highlighted the dire socioeconomic status as a factor constraining their ability to seek care, specifically preventive services. OP tend to under-report their symptoms and mostly seek therapeutic care only at advanced stages. Liang (2022) suggests that promoting patient-centered care can improve the uptake of preventive measures and the management of chronic conditions [48]. Consistent with the wider evidence base [12,14], the findings from this study highlighted the influence of social determinants on OP’s access to PHCCs. The role of family support was highlighted as an essential factor that mitigates OP’s psychological, functional, and cognitive limitations, hindering their ability to seek help, and it also provides them with emotional support and motivation to optimize their well-being. Factors such as limited income, lack of insurance, and low education were reported as barriers to accessing PHC in general. Yet, they appeared to influence the choice of setting in favor of PHCC use. Long travel distances, combined with physical and cognitive declines, make the healthcare-seeking process more difficult. Other factors, including religious and political beliefs, fear, and social roles, manifested a dual effect on access to PHCCs, shaped by the dynamic interplay between demand and supply of services. Similar to findings from LMICs [12], being a woman, living in urban areas, and presenting low-acuity conditions were reported as factors that enable access to PHCCs.

At the healthcare reaching level, several factors related to accommodation, service availability, and OP’s ability to get PHCCs were identified and aligned with the previous evidence base [12]. Kwaitana et al. (2024) highlighted the proximity of centers as an enabler of access for older people, which was particularly important in this study given the increased cost of transportation following the economic downturn. However, limited government funding for PHCCs’ programs impacted the availability, continuity, and quality of services, especially for chronic medication provision programs. Discontinuing medication provision leads to harmful health outcomes, especially since OP cannot afford to purchase medications at pharmacies. They only take medications available at the PHCCs, skip others, omit doses, or make amendments to their prescriptions against their physicians’ advice and international guidelines. Cherfane et al. (2024) assessed the impact of the economic crisis on health-seeking behaviors among Lebanese patients with diabetes and hypertension. They reported that the discontinuation of medications resulted in non-adherence to treatment regimens, dissatisfaction with the health system, and adverse mental outcomes [49]. Moreover, the shortage of family medicine and geriatric specialists, as well as the lack of capacity building in caring for older people, directly affects the appropriateness of care older people receive at PHCCs, contrary to the WHO’s recommendation to promote age-friendly PHCCs [50]. The Syrian crisis and the Lebanese economic recession have increased the demand for services delivered through PHCCs, leading to extensive waiting times at overcrowded facilities. Findings highlight disorganized services as a primary challenge, in addition to advanced age, which is complicated by mobility and functional limitations in older people. Living in urban areas characterized by enhanced transportation infrastructures enables OP’s ability to reach care settings.

Regarding healthcare utilization, the nominal fee for services is one major enabler for OP’s access, especially after the severe economic crisis. However, funding shortages led to the discontinuation of supportive programs. This imposed a higher contribution payment to cover the fees of services and an additional out-of-pocket payment to get services that are no longer available through PHCCs. The absence of social pension plans and the economic crisis increased OP’s financial dependence, fostering a sense of being a burden that undermined their dignity in old age. Gao et al. (2022) suggest that advanced socio-economic statuses are correlated with increased access to PHC in general but decreased use of PHCCs and public services. In Lebanon, the lack of direct government funding for PHCCs has magnified inequities within the system, largely due to disparate funding models.

Regarding healthcare consequences, many factors affect the appropriateness of services and the OP’s ability to engage with care. Several participants reported that high patient loads, limited physician availability, and the current payment system are factors that compromise the quality of clinical examinations, which they described as superficial and focused primarily on the chief complaint. The comprehensive geriatric assessment that can reduce hospitalization among older people at high risk [51] is not being applied systematically across all PHCCs. In contrast to the WHO’s agenda for integrated care for older people (WHO, 2018b), superficial examinations limit the delivery of holistic and comprehensive care, rendering care coordination impossible. The findings highlight that Lebanese PHCCs lack person- and family-centered care, self-management resources, and successful collaborative practice, which were reported as access enablers in a similar Canadian study [52]. Fair remuneration for physicians, care coordination, and comprehensive patient-centered approaches are essential strategies to improve care for older people [53]. Nurses at PHCCs play a pivotal role in patient preparation and education as part of the medical consultation and should be trained and empowered to undertake holistic assessments [54–56]. An effective engagement in the discussion about the OP’s care plans is a shared responsibility among the older person, the informal caregiver, and the care provider.

### Study strengths and limitations

This study presents several major strengths, including the involvement of different stakeholders through gatekeepers, the use of various methods to collect data, effective data protection strategies, and a comprehensive assessment of the access process guided by Levesque et al.’s validated framework. Another strength is the relevance of the findings for policymakers and PHC providers, guiding evidence-based practices. However, the adopted recruitment method limited outreach to people with cognitive limitations and those from very poor backgrounds with limited access to phones. This challenge was addressed by including family members who helped represent their views. There was also a challenge in recruiting more physicians due to workload and in getting them to commit to research activities longer than 10 minutes. Financial limitations and data protection concerns acted as barriers to translating more transcripts into English, which limited the research team’s ability to engage further in data analysis. Limited time and human resources constrained the inclusion of more people benefiting from the services provided by the five PHCCs serving the Koura region. Qualitative methods were employed, and it is acknowledged that the findings are not generalizable and represent the experiences of the participants, which may be similar to those of others.

## Conclusions

Applying Levesque et al.’s framework allowed a comprehensive assessment of factors that affect the OP’s access to PHCCs in Lebanon. The economic crisis exacerbated the OP’s vulnerabilities and accelerated the use of PHCCs, underscoring the urgent need to redesign PHC services to meet the OP’s unique needs. While many identified access barriers and enablers are common globally, others are specific to the Lebanese context especially when it comes to service funding models, the acceptability and affordability of services with people’s ability to seek and pay for services. Enablers and barriers varied across practices and service delivery strategies within PHCCs. Identified barriers mapped across all service dimensions and OP’s attributes indicate that maximizing access to PHCCs is a shared responsibility of several stakeholders and sectors. The role of the MOPH is essential in increasing PHC funding, enhancing healthcare coverage for older people, putting healthy ageing at the top of national agendas, in addition to providing information using methods that the OP and their families can access, including people with lower literacy, who may be unfamiliar with accessing preventive services. Research must support policy makers and practitioners in enhancing evidence-based practices and delivering people-centered care.

## Supporting information

S1 File Topic guides. (DOCX)

S2 File The application of the Framework Method. (DOCX)

S3 File Analytical frameworks. (DOCX)

S4 File Quotes in Arabic translated into English. (DOCX)

S5 File Code classification into categories, subthemes, and themes. (DOCX)

S6 File Mapped summary of access barriers and enablers. (DOCX)
