# Supplementary material for: Barriers and enablers to primary health care center access for older people in Lebanon: A qualitative inquiry
Source: PLoS One. 2025 Oct 23;20(10):e0335073. doi: 10.1371/journal.pone.0335073 (PMC12548930; doi:10.1371/journal.pone.0335073)
Supplement: S1 File — (DOCX) [file pone.0335073.s001.docx]

Supplementary File S1. Topic Guides

Focus group discussions and interviews with older people (users of PHCCs services)

| Guide | Questions | Duration |
| --- | --- | --- |
| Welcome | Welcome participants and thank them for their valuable participation | 2 minutes |
| Introduction | Researcher introduces herself  Study aims  Description of the focus group discussion/interview rules | 5 minutes |
| Warm up | In your opinion, what are PHC services? | 3 minutes |
| Discussion | - Describe your experience with accessing services delivered by PHCCs? What are the challenges to access those services?  Approachability   - In your opinion, how informed are you and older people in general regarding services delivered by PHCCs?   Acceptability   - In your opinion, what are the factors that affect your/older people’s ability to obtain and accept care?   Affordability   - Could you describe how the PHC fees affect your/older people’s behaviors in terms of accessing or using certain services?   Appropriateness   - How do you describe the appropriateness of care delivered to older people in PHCCs? Does care respond to your/their needs? - What is the perceived difference in the care delivered to older people in PHCCs and private clinics?   Ability to perceive:   - In your opinion, what factors may affect your/older people’s perception of need for PHC services?   Ability to seek:   - In your opinion, what factors may affect your/older people’s ability to seek PHC services? - What type of healthcare services/settings do you/older people seek?   Ability to pay:   - In your opinion, what factors impede your/older people’s ability to use and pay for needed PHC services?   Ability to engage:  From your experience, how do you reflect on your/older people’s engagement in decision-making concerning your/their health and healthcare services? Who is involved in this process? | 75 minutes |
| Summary  Finish | Summarize the discussion  Thank the participants for their time and participation  Ask if someone would like to be contacted to take part in the next phase  Adjourn the meeting | 5 minutes |

Focus group discussions and interviews with older people (non-users of PHCCs services)

| Guide | Questions | Duration |
| --- | --- | --- |
| Welcome | Welcome participants and thank them for their valuable participation | 2 minutes |
| Introduction | Researcher introduces herself  Study aims  Description of the focus group discussion/interview rules | 5 minutes |
| Warm up | How do you describe the PHC services? | 3 minutes |
| Discussion | - Have you ever had an experience with access to a PHCC? If yes describe, and if not, why?  Approachability   - In your opinion, how and to what extent are you and older people informed in general about services delivered by PHCCs?   Acceptability   - In your opinion, what are the factors that affect your/older people’s ability to obtain and accept care?   Affordability   - Could you describe how the PHC fees affect your/older people’s behaviors in terms of accessing or using PHC services?   Appropriateness   - Can you describe the appropriateness of care delivered to older people in PHCCs? Does care respond to your/their needs? - What about the services you are getting in the facility that you visit? is there a perceived difference in the care delivered to older people in PHCCs and private clinics?   Ability to perceive:   - In your opinion, what factors may affect your/older people’s perception of need for PHC services?   Ability to seek:   - In your opinion, what factors may affect your/older people’s ability to seek PHC services? - What type of healthcare services/settings do you/older people seek?   Ability to pay:   - In your opinion, what factors impede your/older people’s ability to use and pay for needed PHC services?   Ability to engage:  From your experience, how do you reflect on your/older people’s engagement in decision-making concerning your/their health and healthcare services? Who is involved in this process? | 75 minutes |
| Summary  Finish | Summarize the discussion  Thank the participants for their time and participation  Ask if someone would like to be contacted to take part in the next phase  Adjourn the meeting | 5 minutes |

Focus group discussions and interviews with family members of older people

| Guide | Questions | Duration |
| --- | --- | --- |
| Welcome | Welcome participants and thank them for their valuable participation. | 2 minutes |
| Introduction | Researcher introduces herself  Study aims  Description of the focus group discussion/interview rules | 5 minutes |
| Warm up | - How do you define PHC services? | 3 minutes |
| Discussion | - Have you ever had an experience with access to a PHCC? If yes describe, and if not, why?  Approachability   - In your opinion, how and to what extent are you and older people informed about services delivered by PHCCs?   Acceptability   - In your opinion, what are the factors that affect older people’s ability to obtain and accept care?   Affordability   - Could you describe how the PHC fees affect your behaviors and those of older people in terms of accessing or using certain services?   Appropriateness   - How do you describe the appropriateness of care delivered to older people in PHCCs? Does care respond to their needs? - What is the perceived difference in the care delivered to older people in PHCCs and private clinics?   Ability to perceive:   - In your opinion, what factors may affect older people’s perception of need for PHC services?   Ability to seek:   - In your opinion, what factors may affect older people’s ability to seek PHC services? - What type of healthcare services/settings do older people and you, as caregivers, seek in your community?   Ability to pay:   - In your opinion, what factors impede the ability of older people and your ability, as caregivers, to use and pay for needed PHC services?   Ability to engage:   - From your experience, how do you reflect on older people’s engagement in decision-making concerning their health and healthcare services? Who else is involved in this process? | 75 minutes |
| Summary  Finish | Summarize the discussion  Thank the participants for their time and participation  Ask if someone would like to be contacted to take part in the next phase  Adjourn the meeting | 5 minutes |

Focus group discussions with service providers

| Guide | Questions | Duration |
| --- | --- | --- |
| Welcome | Welcome participants and thank them for their valuable participation. | 2 minutes |
| Introduction | Researcher introduces herself  Study aims  Description of the focus group discussion/interview rules | 5 minutes |
| Warm up | - How do you define PHC services? | 3 minutes |
| Discussion | - What is your experience with delivering PHC services for older people within PHCC? What are the challenges related to providing PHC for older people within PHCCs?  Approachability   - In your opinion, how informed older people are regarding services delivered by PHCCs?   Acceptability   - In your opinion, what are the factors that affect older people’s ability to obtain and accept care?   Affordability   - Could you describe how the PHC fees affect older people behaviors in terms of accessing or using certain services?   Appropriateness   - How do you describe the appropriateness of care delivered to older people in PHCCs? Does care respond to their needs? - What is the perceived difference in the care delivered to older people in PHCCs and private clinics?   Ability to perceive:   - In your opinion, what factors may affect older people’s perception of need for PHC services?   Ability to seek:   - In your opinion, what factors may affect older people’s ability to seek PHC services? - What type of healthcare services/settings do older people seek in your community?   Ability to pay:   - In your opinion, what factors impede the ability of older people to use and pay for needed PHC services?   Ability to engage:   - From your experience, how do you reflect on older people’s engagement in decision-making concerning their health and healthcare services? Who is involved in this process? | 75 minutes |
| Summary  Finish | Summarize the discussion  Thank the participants for their time and participation  Ask if someone would like to be contacted to take part in the next phase  Adjourn the meeting | 5 minutes |
