# Supplementary material for: Barriers and enablers to primary health care center access for older people in Lebanon: A qualitative inquiry
Source: PLoS One. 2025 Oct 23;20(10):e0335073. doi: 10.1371/journal.pone.0335073 (PMC12548930; doi:10.1371/journal.pone.0335073)
Supplement: S2 File — (DOCX) [file pone.0335073.s002.docx]

**Supplementary File S2. The application of the Framework Method**

The Framework Method, is applied to analyze data through seven iterative stages, as presented by Gale et al., (2013). This thematic analysis approach is aligned with the pragmatic research paradigm as it sits in the middle on the continuum between the reflexive thematic analysis that fully incorporates qualitative research values and the coding reliability thematic analysis that follows neopositivists objectivity standards [1]. Being widely applied in policy and applied health research [2] and commonly used to analyze semi-structured interviews [3], it fits to answer this study research question. It serves to systematically analyze the data by adopting a combined coding approach (deductive and inductive) as this study is guided by the Patient-Centered Access to Health Care framework [4], then interpret data through comparing and contrasting data per theme across several participants [3]. This framework that served to elaborate the topic guides for data collection will also be used to guide data analysis and will be refined, by identifying contextual factors affecting the access of older people to primary health care centers in Lebanon. Data analysis and interpretation happened through the following non-linear process.

- **Stage 1: Data transcription**

Concurrently to data collection [*5*], the same researcher (SD) who was conducting interviews and FGDs was manually transcribing the audio-recordings verbatim for data protection purposes. Line numbers and text boxes were inserted to the widened margins of each transcript. Left boxes were dedicated to assign codes and themes while right boxes were used to add summaries and notes.

- **Stage 2: Familiarization with the data set**

Collecting data and transcribing audio-recordings manually by the same author who conducted the analysis and re-read the manuscripts while checking the audio-recordings offered a great opportunity to get immersed within the data and to make early impressions and document notes. Those impressions were shared and discussed regularly with the research team members informing adequate decisions.

- **Stage 3&4: coding and development of the analytical framework**

This study is guided by the Patient-Centered Access to Health Care framework [4]. This framework informed the elaboration of topic guides for data collection and guided the data analysis. Prior to this study, the framework was adapted to the current topic through a scoping review that examined barriers and enablers to access of older people to PHC in Low- and middle-income countries [6]. This refined version provided a pre-determined set of themes and codes to explore through participants accounts (initial framework presented in Supplementary file 3). However, a combined approach to coding was adopted; in addition to the deductive coding by applying the predetermined set of codes and themes, other semantic and latent codes capturing unexpected aspects of participants’ experiences [3] were inductively generated and the analytical framework adapted. Codes were described as per the framework and also as per participants’ accounts (new codes described only through participants’ accounts). At the early coding stages, SD and TK have coded independently three transcripts translated into English for this purpose, then they met to discuss their perspectives and to ensure that all relevant aspects are captured. Discussions were instrumental to refine the working analytical framework that was iteratively updated to include all new themes (the refined analytical framework is presented in Supplementary file 3). The analytical framework was never final until the last manuscript. Under each sub-theme (dimensions or abilities) codes were categorized as presented in Supplementary file 5. Regarding semantic and latent codes, “fear” is provided as an example of semantic codes as “fear from physicians and medical procedures” was literally reported by participants as a barrier to seeking care. “Perceived lack of support” is an example of latent codes used to interpret older people’s accounts while comparing their life conditions to those of older people living abroad.

- **Stage 5: Application of the analytical framework**

Relevant sentences and passages were underlined then appropriate codes assigned manually in left boxes while notes, summaries, and quotes were added to the right boxes (as shown in figure 1). The analytical framework was refined according to new codes generated from participants’ accounts. Refinements consisted either of adding new codes/themes or amending the descriptions of an existing code/theme. For example, the description of “fear” evolved throughout the analysis as participants used this term to report different things like “fear from physicians and medical procedures” and “fear of discovering a disease or malfunction” and “fear of revealing a condition that requires care that they cannot afford”. Different nuances were captured with the “fear” description. Once the last manuscript is analyzed a final refinement is required to revisit codes and categories.

- **Stage 6: Data charting**

The framework matrix consisted of several Excel spread sheets featuring each a main theme of the analytical framework. Columns represented codes while rows represented participants designated by their pseudonyms. Referenced elements in the right margins (interpretations and codes) were then charted appropriately into the framework matrix (example shown in figure 2).

- **Stage 7: Data interpretation**

Data collected through FGDs and individual interviews are analyzed as one data set. Charted data allowed the research team to make interpretations through comparing and contrasting through diverse accounts, then summarizing and synthesizing elements illustrated under each code across all participants. Data were classified per themes that are relevant to practice [7]; presented data define the PHC concept as perceived by participants and explain their experiences and factors influencing their access to PHCCs [2]. An expert translator and A Lebanese-American friend who is English native speaker assisted SD to translate the quotes that are reported in both languages (Supplementary file 3).

**Figure A. Example of coded extract**


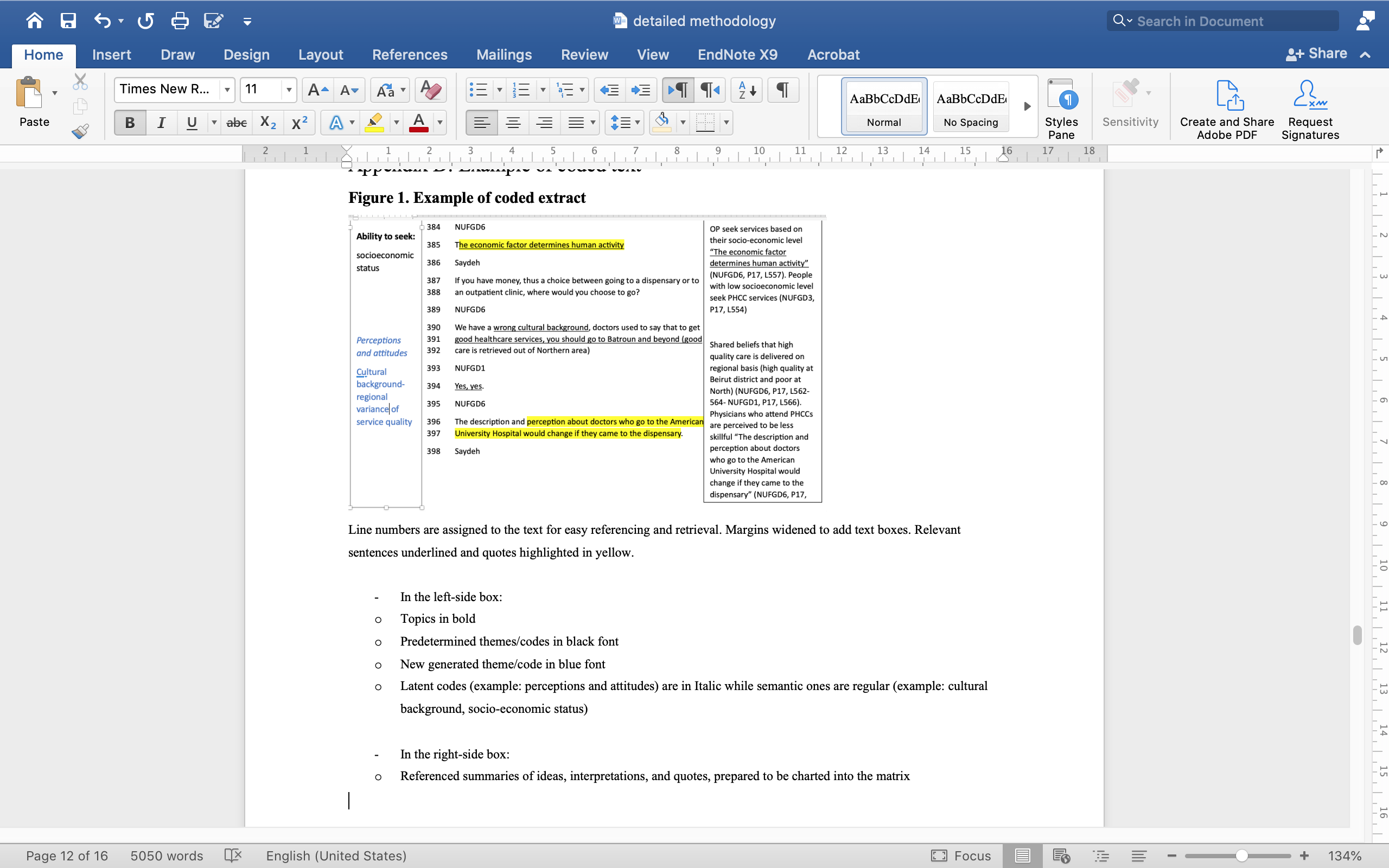


**Figure B. Example of a framework matrix sheet**

**
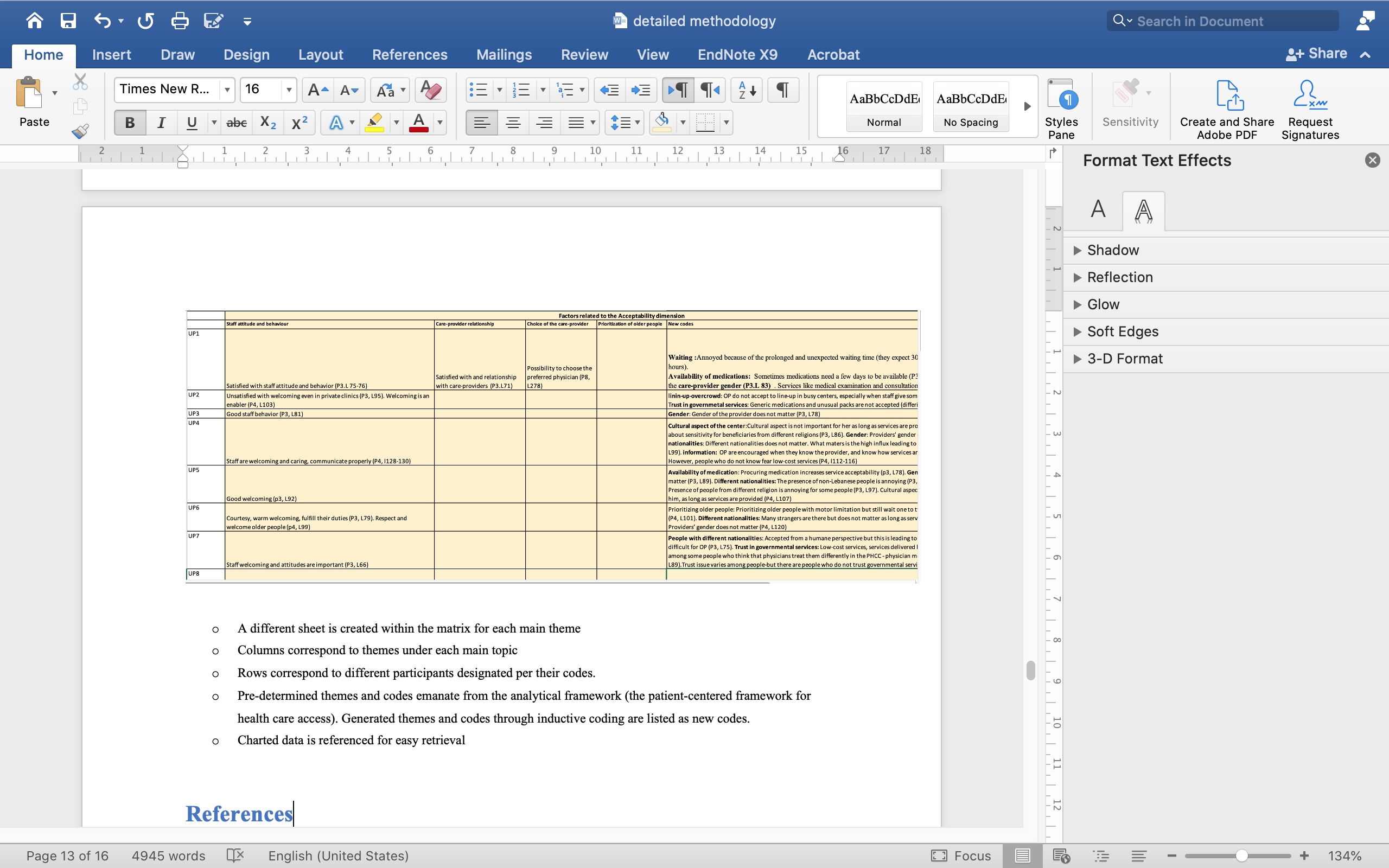
**

# References

1. Braun V, Clarke V. One size fits all? What counts as quality practice in (reflexive) thematic analysis? Qualitative Research in Psychology [Internet]. 2021; 18(3):[328-52 pp.]. Available from: <https://doi.org/10.1080/14780887.2020.1769238>.
2. Ritchie JaS, L. . Qualitative Data Analysis for Applied Policy Research.

In: Alan Bryman BB, editor. Analyzing Qualitative Data. Routledge, London: taylor and Francis Group; 1994. p. 173-94.

3. Gale NK, Heath G, Cameron E, Rashid S, Redwood S. Using the framework method for the analysis of qualitative data in multi-disciplinary health research. BMC Medical Research Methodology [Internet]. 2013; 13(1):[117 p.]. Available from: https://doi.org/10.1186/1471-2288-13-117.

4. Levesque J-F, Harris MF, Russell G. Patient-centred access to health care: conceptualising access at the interface of health systems and populations. International Journal for Equity in Health [Internet]. 2013; 12(1):[18 p.]. Available from: https://doi.org/10.1186/1475-9276-12-18.

5. Creswell J. Research design: Qualitative, Quantitative and Mixed Methods Approaches: Sage Publications; 2009. Available from: https://www.ucg.ac.me/skladiste/blog_609332/objava_105202/fajlovi/Creswell.pdf.

6. Dableh S, Frazer, K., Stokes, D., & Kroll, T. . Access of older people to primary health care in low and middle-income countries: A systematic scoping review. . PLOS ONE [Internet]. 2024; 19(4). Available from: https://journals.plos.org/plosone/article?id=10.1371/journal.pone.0298973.

7. Kelly LM, Cordeiro M. Three principles of pragmatism for research on organizational processes. Methodological Innovations. 2020;13(2):2059799120937242.
