# Supplementary material for: Barriers and enablers to primary health care center access for older people in Lebanon: A qualitative inquiry
Source: PLoS One. 2025 Oct 23;20(10):e0335073. doi: 10.1371/journal.pone.0335073 (PMC12548930; doi:10.1371/journal.pone.0335073)
Supplement: S3 File — (DOCX) [file pone.0335073.s003.docx]

**Supplementary File S3. Analytical frameworks**

**Initial analytical framework**

| Code | Description |
| --- | --- |
| Definition of PHC | |
| Included services | Any kind of services that constitute primary care; they include preventive care, therapeutic care, rehabilitation, and palliative care |
| Barriers and enablers | |
| Approachability | It means that potential users have awareness about available services and their eligibility to use them. This related to transparency, information, and outreach activities |
| Information on available services | Type of information about PHCCs (localities, delivered services, eligibility criteria) and mode of dissemination of this information (word of mouth, information campaigns, community meetings, etc.) |
| Acceptability | It relates to cultural and social factors determining the possibility for people to accept the aspects of the service. It’s linked to care providers’ sex, social groups, and cultural competence, and people’s experience of trust, respect, discrimination, and inclusiveness. |
| Staff attitude and behaviour | The way the staff members welcome and communicate with older people including respect, warmth, and consideration |
| Care-provider relationship |  |
| Choice of the care-provider | The possibility to choose the care provider they prefer |
| Prioritization of older people | Considering older people as a priority and give them a pass when line up or waiting to get services |
| Affordability | Relates to the economic capacity for people to spend resources and time to use appropriate services. It is a balance between the service cost and the user’s resources including financial coverage |
| Allocated funds for geriatric care | Funding schemes and sources dedicated to finance geriatric services and programs |
| Transportation and service cost | Amount that people should pay to commute to the PHCC and to get services |
| Contribution fees | The amount of money that the beneficiary should pay to contribute to fulfilling a service fee |
| Appropriateness | Highlights the fit between service and need. It consists of delivering people-centered care in an appropriate way. It emphasizes the identification of unique needs and the delivery of responsive services |
| Geriatric clinical examination quality | Describes timeliness and duration of the clinical examination, geriatric assessment, correct treatment, technical and interpersonal quality of the services |
| Client-provider relationship | Service providers are expected to create an environment where clients feel respected and enable them to share concerns with service providers without hesitation. This includes multiple components such as maintaining privacy, keeping clients informed, follow-up care, giving appropriate time and attention, and addressing and respecting the client’s concerns and choices. |
| Care comprehensiveness | Full range of health services including preventative and therapeutic care addressing the physical, mental, and cognitive health needs of the person. |
| Care coordination | The deliberate organization of patient care activities between two or more participants (including the patient) involved in a patient's care to facilitate the appropriate delivery of health care services. |
| Care continuity | The process of ongoing care delivery without interruption. |
| Ability to perceive | This is the ability to perceive the need for care among older people |
| Literacy | The ability to read and write |
| Age | Is the state of being old or the process of becoming older |
| Need | Refer to health needs like pain and any acute or chronic conditions that require care |
| Income | Money received on regular basis for work or through investments |
| Use and preference | Relate to the people’s habits or likeability to go to a specific care provider or setting |
| Ability to seek | Relates to the concepts of personal autonomy and capacity to choose to seek care, knowledge about health care options and capability to take actions to obtain health care |
| Religion | Refers to the person’s religious beliefs |
| Race | Refer to ethnic groups |
| Socio-economic status | Social standing or class describing people based on their education, income, and type of job. Socioeconomic status is usually described as low, medium, and high. |
| Cognitive condition | Person's ability to think, learn, remember, use judgement, and make decisions |
| Ability to pay | Describes the capacity to generate economic resources - through income, savings, borrowing, insurance plans - to pay for health care services without catastrophic expenditure |
| Income | Money received on regular basis for work or through investments |
| Health insurance | Agreement in which an insurance company agrees to pay for some or all person’s medical expenses in exchange for a monthly premium payment. |
| Ability to engage | Ability of older people to participate and get involved in shared decision making about treatment and care plans. It requires a set of skills like health literacy, self-efficacy, and ability to communicate. |
| Empowerment | People having freedom and power to do what they want and control over their own lives |
| Information | Knowledge that a person has about his condition |
| Adherence |  |
| Caregiver support | The availability of a family member who regularly assists the older person and looks after him/her |
| Other generated codes | |
|  |  |
|  |  |
|  |  |
|  |  |
|  |  |
|  |  |

**P.S** this analytical framework draws on the below conceptual framework for the access of older people to PHC in LMICs (Dableh et al., 2024)-Adapted from the Patient-Centered Access to Health Care framework presented by Levesque et al (2013)


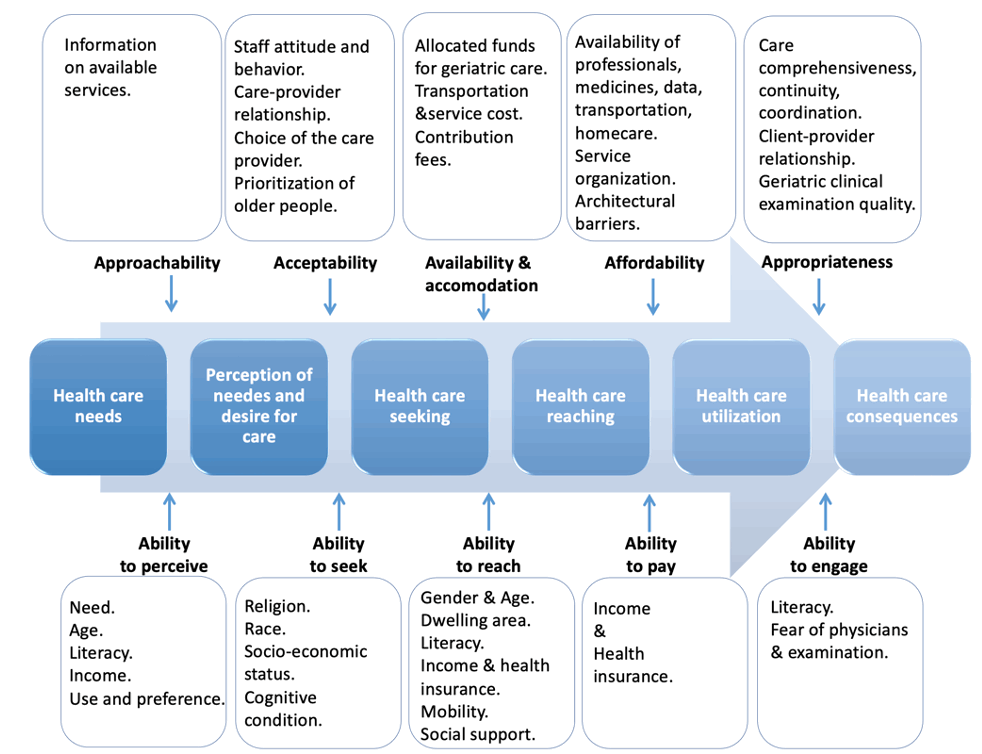


**Final analytical framework**

This framework includes two descriptions for several codes: the first as explained through the adapted conceptual framework of Levesque et al. (second column), and the second as described through participants’ accounts (third column). New codes generated from participants’ accounts present only one description (third column).

**Theme 1: Perception of needs and desire for care**

**1.1 Approachability**

| **Code** | **Description as per Levesque’s adapted framework** | **Description as per participants accounts** |
| --- | --- | --- |
| Category 1.1.1. Information on available services | | |
| Lack of knowledge on PHC |  | Refers to the inability of participants to list services included within the PHC package correctly. |
| Lack of knowledge on PHCCs |  | Refers to the inability of participants to recall information about PHCCs (localities, delivered services, eligibility criteria). |
| Mixing between PHCCs and dispensaries |  | Refers to participants using PHCCs and dispensaries interchangeably without being able to identify the difference between the two types of settings. |
| Category 1.1.2. Source of information | | |
| Informal channels |  | Includes informal or non-intentional mode of information dissemination like word of mouth and screening campaigns. |
| Formal channels |  | Includes formal strategies to disseminate information about PHC services like information campaigns, community meetings, and outreach activities. |

**1.2 Ability to perceive**

| **Code** | **Description as per Levesque’s adapted framework** | **Description as per participants accounts** |
| --- | --- | --- |
| Category 1.2.1. Socio-demographic determinants | | |
| Education and health literacy | The ability to read and write, process and use health information. | While education describes the personal educational level, health literacy refers to the degree to which individuals can process and use health information. |
| Age | Is the state of being old or the process of becoming older. | Refers to getting older. |
| Living arrangement |  | Describes whether living alone or with a family. |
| Socioeconomic status |  | Refers to the ability to afford care services. |
| Category 1.2.2. Health determinants | | |
| Health needs | Refer to health needs like pain and any acute or chronic conditions that require care. | Need encompasses mental, functional and physiologic issues and refers to any perceived symptoms, malfunction, weakness, pain, anxiety, and chronic disease management. |
| Emotional status |  | Refers to stress and fear. Fear can describe being afraid of doctors, medical procedures, or detecting physical malfunction OR fear of death and concern even of simple symptoms. |
| Cognitive abilities |  | Describes the capability of making decisions and verbalizing needs. |
| Category 1.2.3. Service-related determinants | | |
| Free services |  | Refer to provision of free of charge services like campaigns. |

**Theme 2: Healthcare seeking**

**2.1 Acceptability**

| **Code** | **Description as per Levesque’s adapted framework** | **Description as per participants accounts** |
| --- | --- | --- |
| Category 2.1.1. Provider-related determinants | | |
| Staff behavior and attitude | Staff members conduct with beneficiaries including respect, structural ageism, trust in nurses, prioritization of OP, and interpersonal relationships. | The way the staff members welcome and communicate with older people including respect, warmth, care, and consideration. |
| Provider’s characteristics | Service providers are expected to create an environment where clients feel respected and enable them to share concerns with service providers without hesitation. | Refer to providers’ consciousness, character, values, skills, and experience. It also includes the possibility to choose the care provider. |
| Negative role of physicians |  | Refers to physicians discouraging people from suing PHCCs services for being of a bad quality. |
| Category 2.1.2. Service-related determinants | | |
| Service organization | ? | Refers to the consequences of arrangements decided by the PHCC concerning the service delivery like waiting time, overcrowd, lining-up, and prioritization of older people. |
| Availability and quality of services |  | Availability denotes the scope of services delivered through PHCCs, considering OP’s health needs, and its influence on OP’s help seeking decisions. Quality of services describes the perceived quality of services notably the medications and clinical examinations and its influence on OP’s help seeking decisions. |
| Information about services |  | Refers to information made available especially in relation to service provision, funding source, and target population. |
| Category 2.1.3. Social and cultural determinants | | |
| Trust in governmental services |  | Attitude that people have toward public services or services provided by the government even through the private sector. Denotes also the trust toward physicians and nurses. |
| Cultural aspect of the center |  | Refers to religious, political, or any specific affiliation of the centre. |
| Influx on non-Lebanese beneficiaries |  | Refers to the massive number of non-Lebanese beneficiaries who seek PHCC services especially after the Syrian crisis. |
| Shared experiences |  | Lived experiences and impressions that users of PHCCs services build and share with other society members. |
| Critical negative perception |  | Describes shared cultural beliefs and attitudes affecting negatively the decision of using PHCCs services. This includes labelling PHCCs users as disadvantaged, correlation between low cost and low quality and perceived perception of taking poor people’s advantages by using the PHCCs. |

**2.2 Ability to seek**

| **Code** | **Description as per Levesque’s adapted framework** | **Description as per participants accounts** |
| --- | --- | --- |
| Category 2.2.1. Socio-demographic determinants | | |
| Socioeconomic status | Refers to the social standing or class describing people based on their education, income, and type of job. | Describes the socio-economic status in relation to PHC use patterns and choice of healthcare settings. |
| Gender |  | Highlights the relation between gender and help seeking behaviours. |
| Education and health literacy |  | While education describes the personal educational level, health literacy refers to the degree to which individuals can process and use health information. |
| Category 2.2.2. Social and cultural determinants | | |
| Family and social support |  | Refers to the presence of family members and people who look after the older person’s health. |
| Roles and functions |  | Highlights the role and occupation that a person should fulfil on a daily basis in relation to health seeking behaviours. |
| Personal beliefs |  | Beliefs that older people have about PHCCs and their services leading to attitudes toward seeking them or not like trust of services, use and preference (people’s habits or likeability to go to a specific care provider or setting). |
| Religion | Describes religious beliefs in relation to PHC use. | Highlights religious affiliation affecting health seeking decisions and the choice of care setting. |
| Category 2.2.3. health determinants | | |
| Emotional status |  | Describes how OP feel like being sad, depressed, eager to live more, or feeling like a burden. It also describes fear of contamination during pandemics, fear of doctors or medical procedures, or fear of detecting physical malfunction. |
| Physical abilities |  | Denotes the ability to perform instrumental activities of daily living autonomously like managing money, making phone calls, and driving among others. |
| Health needs |  | Refers to health needs like pain and any acute or chronic conditions that require care. |
| Category 2.2.4. Environmental determinants | | |
| Travel distance |  | Refers to the travel time and distance to reach the nearest PHCCs. |
| Transportation |  | Describes the availability of adequate and affordable transportation system to facilitate the access to PHCCs. |

**Theme 3: Healthcare reaching**

**3.1 Availability and accommodation domain**

| **Code** | **Description as per Levesque’s adapted framework** | **Description as per participants accounts** |
| --- | --- | --- |
| Category 3.1.1. Service-related determinants | | |
| Availability of professionals | Denotes the presence of the health professionals and their qualifications. | Refers to the adequacy of the number of professionals operating at the PHCCs, their capacity building on geriatric issues, and their schedule (number of hours and days per week). |
| Availability of medications and equipment | Describes the availability of sufficient and adequate resources to produce services. | Refers to the existence of needed medications and equipment like assistive devices at PHCCs. |
| Scope of services | Refers to the existence of health resources. | Refers to the existence of needed services at PHCCs and the delivery of home-based services. |
| Service organization | Appointment mechanism, opening hours, waiting time, waiting areas | Refers to arrangements decided by the PHCC concerning the service delivery and their consequences including waiting time, possibility to book appointments over the phone, assistive personnel, and physician’s payment system. |
| Category 3.1.2. Environmental determinants | | |
| Travel and transportation | Refers to the distribution of services and transportation system. | Refers to the travel time and distance to reach the nearest PHCCs and to the availability of adequate and affordable transportation system to facilitate the access to PHCCs. |
| Built environment | Describes building accessibility | Denotes accessibility and physical structures that either enable or constrain access like existence of ramps, lifts, stairs, etc. |

**3.2 Ability to reach**

| **Code** | **Description as per Levesque’s adapted framework** | **Description as per participants accounts** |
| --- | --- | --- |
| Category 3.2.1. Socio-demographic determinants | | |
| Age | Refers to advanced age. | Refers to getting older. |
| Dwelling area | Refers to urban or rural context and consequences on health seeking behaviors. | Refers to urban or rural context and consequences on health seeking behaviors. |
| Category 3.2.2. Health determinants | | |
| Mobility and assistive devices | Highlights limited mobility and physical disability. | Highlights motor limitations and denotes the availability of assistive equipment that facilitates the walk-in of people with limited mobility to the PHCCs |

**Theme 4: Healthcare utilization**

**4.1 Affordability**

| **Code** | **Description as per Levesque’s adapted framework** | **Description as per participants accounts** |
| --- | --- | --- |
| Category 4.1.1. Economic determinants | | |
| Availability of geriatric funds | Funding schemes and sources dedicated to finance geriatric services and programs | Refers to the funding support provided through local or external entities to run the PHCCs and geriatric programs and provide resources like chronic medications. |
| Transportation cost | Amount that people should pay to commute to the nearest PHCC. | Amount that people should pay to commute to the nearest PHCC. |
| Category 4.1.2. Service-related determinants | | |
| Service fees |  | The precised amount that people should pay to get the service. It covers the contribution of clients and the specific considerations applied for people who cannot afford the service cost. |
| Contribution fees | Refers to the amount that people should contribute to complete the fees of services and get them. | Covers the specific considerations applied for people who cannot afford the service cost. |

**4.2 Ability to pay**

| **Code** | **Description as per Levesque’s adapted framework** | **Description as per participants accounts** |
| --- | --- | --- |
| Category 4.2.1. Socio-demographic determinants | | |
| Socioeconomic status | Describes income in relation to health care use patterns. | Describes general financial abilities resulting from the income, savings, nature of the previous work and other financial resources (donation from a family member or else). |
| Insurance and pension plans |  | Describes the availability or lack of insurance or pension plans in relation to health seeking behaviours. |
| Familial and social support |  | Refers to the contribution of family members or social groups to cover service fees. |
| Category 4.2.2. Economic determinants | | |
| The economic crisis |  | Describes the consequences of the economic crisis that hit the country at 2019 which is characterized by a dysfunction of national insurance funds, a currency devaluation, and a bank system failure with major consequences on health seeking behaviours. |

**Theme 5: Healthcare consequences**

**5.1 Appropriateness**

| **Code** | **Description as per Levesque’s adapted framework** | **Description as per participants accounts** |
| --- | --- | --- |
| Category 5.1.1. Service-related determinants | | |
| Geriatric clinical examination | Describes timeliness and duration of the clinical examination, geriatric assessment, correct treatment, technical and interpersonal quality of the services | Refers to the duration and the quality of the examination. It also denotes the comprehensive geriatric assessment and the patient preparation. |
| Care coordination | Describes the deliberate organization of patient care activities between two or more participants (including the patient) involved in a patient's care to facilitate the appropriate delivery of health care services. | Refers to the factors that constrain care coordination and to the role of family members in bridging coordination gaps. |
| Care continuity | Refers to the process of ongoing care delivery without interruption. | Refers to the factors that constrain care continuity notably the provision of chronic medications. |
| Category 5.1.2. Provider-related determinants | | |
| Care comprehensiveness | Designate the full range of health services including preventative and therapeutic care addressing the physical, mental, and cognitive health needs of the person. | Highlights the underlying factors leading to the focus on therapeutic care rather than preventive care notably health education. |
| Client-provider relationship | Service providers are expected to create an environment where clients feel respected and enable them to share concerns with service providers without hesitation. | Refers to factors that affect building a trustful and respectful relationship with providers including the provider’s communication skills, care, and conduct. |

**5.2 Ability to engage**

| **Code** | **Description as per Levesque’s adapted framework** | **Description as per participants accounts** |
| --- | --- | --- |
| Category 5.2.1. Socio-demographic determinants | | |
| Education and health literacy | The ability to read and write | Describes the educational level of the beneficiary and the degree to which individuals can process and use health information. |
| Trust |  | Refers to people who believe that physicians are knowledgeable and accountable for their decisions. |
| Self-efficacy and interest |  | Designates the trait of personality when people dare to ask and know about their care plan. Refers also to some OP who are hopeless and forced by the family members to get care. Interest refers to the benefits that the beneficiary can get out of engaging. This could be knowledge increase or getting cheaper procedure or medication. |
| Category 5.2.2. health determinants | | |
| Cognitive and sensory abilities |  | Describes the presence or absence of any cognitive limitation that challenge the ability to understand and converse. It includes awareness of own’s health status and visual and hearing limitations. |
| Emotional status |  | Describes how OP feel like being sad, depressed, eager to live more, or feeling like a burden. It also describes fear of contamination during pandemics, fear of doctors or medical procedures, or fear of detecting physical malfunction. |
| Category 5.2.3. Provider-related determinants | | |
| Providers’ conduct |  | Refer to providers’ consciousness, character, values, morals, and care. |
| Providers’ communication skills |  | Describes the provider’s verbal and non-verbal communication including active listening. |
| Category 5.2.4. Social and cultural determinants | | |
| Role of family members |  | Refers to when Family members assist OP and engage to seek information and discuss OP’s care plans. |
| Alternative source of information |  | Refers to sources where people get information, rather than care providers, like Google search engine. |
| Category 5.2.5. Service-related determinants | | |
| Examination time and fees |  | Time that the person spends at the physician’s clinic for examination |
