# Supplementary material for: Barriers and enablers to primary health care center access for older people in Lebanon: A qualitative inquiry
Source: PLoS One. 2025 Oct 23;20(10):e0335073. doi: 10.1371/journal.pone.0335073 (PMC12548930; doi:10.1371/journal.pone.0335073)
Supplement: S4 File — (DOCX) [file pone.0335073.s004.docx]

Supplementary File S4. Quotes in Arabic translated into English

Theme 1: Perception of needs and desire for care

|  | Arabic (Lebanese) quotes | English translation |
| --- | --- | --- |
| 1.1 Approachability | في نقص معلومات قوي، معلومات صحيّة، على مستوى الفرد والمجموعة.  انا هي اول مرة بسمع بهيدا البرنامج، بالرعاية الصحية الأولية.  كونا العالم ما بتعرف كيف الادوية والطعومة بتنحفظ، هيدا عائق بوصّل لإنعدام الثقة.  اللاجئين مفكرين إنّو الخدمات لللبنانيين واللبنانيين مفكرين إنن للسوريين. | There is a huge lack in information, health information, at the individual and group levels” (NUP, male, 71-79 age group).  “This is the first time we hear about this program, about primary care” (NUP, male, 71-79 age group).  “The lack of information on how medication and vaccines are conserved is a barrier leading to lack of trust” (FMNU, female, age 43)    “Refugees think services are for Lebanese and Lebanese people think they’re for refugees” (SP, up to 10 years of experience) |
| 1.2 Ability to perceive | اختي كلو بتعرفو بس مش متعلمة ، صار عندا خبرة.  ٩٩٪ من الشعب اللبناني بيتحكّم عآخر نفس.  متل السيارات، لحتي ما عود تمشي.  بحس أيام بدن يجوا يحكوا، بحبوا يجوا يحكوا نحنا منسمعلن.  نوع من الخوف، إنه يدخلوا مثلا عالمستشفى ويصير بدنا وبدنا، وفي ناس مثلا مادتي ما بتسمح لي إني فوت عمستشفى فبلا ما أتحركش بهالشي، بحدفوا.  بخاف من الحكيم ممكن الكبار السن بخافوا من الموت بخافوا من الحكمة.  بيرفضوا، بقلّك خليني موت بلا ما أعرف، إذا عندي شي ورم.  ما بدي روح اعمل فحص، خليني ما بعرف شو في احسن | “My sister knows everything, she’s not educated but she has experience” (FMU, female, 51-65 age group)  “99% of Lebanese people seek care at a final stage” (NUP, male, 60-70 age group)  “Like cars, until they turn off” (NUP, male, ≥80 age group)  “I feel that sometimes they need to talk, they like to come and talk, we listen to them” (SP, up to 10 years of experience)  “They fear going to the hospital and discover the need for further care; some people cannot afford the hospitalization cost, that’s why they prefer skipping explorations, they postpone investigations” (UP, female, 60-70 age group)  “Older people fear physicians, they fear death, they fear medical procedures” (FMU, female, 51-65 age group)  “They refuse and say let us die without knowing, in case we have a tumor” (SP, up to 10 years of experience)  “He would say I don’t want to do the checkup, I prefer not to know” (FMNU, female, 41-50 age group) |

Theme 2: Healthcare seeking

|  | Arabic (Lebanese) quotes | English translation |
| --- | --- | --- |
| 2.1 Acceptability | لأنه بالذهنية تبعنا إنه مركز الرعاية، للفقرا وللكذا بخلوه الموظفين بطريقة التعامل معهم والاستقبال يغير فكره، شي طبيعي.  لما بروح على المستشفى فيه مين يتأهل فيني. لما روح على مستوصف حسيتها عم تربحني من نية.  لمّا بيكون عاجق المركز ما بفوت، حقيقة، ما بقدر.  أنا مرا كبيري وأنا عندي اوجاع ضهر ومريضة، ما فيني روح اقعد تلات أربع ساعات، مصلوبة عكرسي تا فوت عند الحكيم! هذا شيء عم بيعطل علينا انا نجيب الدوا، نحنا بحاجة للدو.  واحد أنا بدي 4 و5 ساعات ما بيجي دوري عند الحكيم، وأمي مرأة كبيرة تاركتها وحدها ختياره.  ومجرد ما شافو عالكرسي هوي ما بينطر ولا دقيقة.  هالشي بيرجع لثقافة الشخص والمعلومات يلّي عندو ياها عن تمويل الخدمات.  كبار السنّ بيتشجّعوا لمّا بيعرفوا مين الحكيم، كيف الخدمات عم تتموّل، وليش سعرن الخدمة قليل.  معلومات عامة انو اذا رحتي عمراكز رعاية صحية أولية تابعة لوزارة الصحة ما في عالاستقبال اللائق، لان بيعتبرو يلبنان انو كل شي تابع للدولة، كل شي "فري" منو كتير خدماتو على المستوى المطلوب، كرمال هيك بخافو الناس يروحوا ع مراكز رعاية صحية أولية.  مفهوم الرعاية الصحية الأوليّة بيتغلّب على الطائفيّة.  لأنّو المركز عندو هالطابع الديني، انا ما بروح عليه.  هيدي مراكز حزبية بدك تصيري، إنتي ملزمة إذا رحتي لعندن إنك ترجعي تنتخبين.  انا ما بحب مثل ما عم بيقولوا، أنا إذا شي حزبي كثير أنا بحس إنه ما بقبلو، ما بفوت. ما بقبل يعني، بينما من غير دين، ما عندي مشاكل  لمّا بشوف هالإشيا بتردّد، بعدّ للعشرة قبل ما روح، وبقول شو إلي شغل انا هونيك، كلّن أغراب.  بفضل ما مروح. بفل أكيد، أكيد.  صاروا اللبنانيي أقليات بين كلّ هالأغراب  وقت بيجي لهون بلاقي هالعجقة وهالخفقة وناس مش لبنانيين كمان عم بياخذوا دوا بيخلي يكون صورة أنه مركز الرعاية منو لائق لالي. ما بيعود يجي.  أيام بقولولنا نحنا عم نحكمكن عحسابنا.  بتحسيهم بنظرات عينيهم يعني هيك.  تيجى عالصيت كل حدا يبعت ويجي يعني ابسطوا من المعاملة، المعاملة مش فردية على شخص، كل إنسان بيطلع نفس الاستقبال نفس المعاملة.  حسب السمعة، حسب الأشخاص شو عم بيقولو  هني يلي عم بيروحوا ما عم يلاقوا دوا، شو بدي روح اعمل  أنا بعرف كتير ناس بفكروا إنو هيدا للفقرا بس من هيك ما بيروحوا  بقول خلّي العالم يلّي مش مضمونين حرام، يستفيدوا من خدمات المركز.  العالم بيشوفوا إنو إذا واحد راح عالمركز الصحّي كأنو هالشي بنزّل من مقامو.  بيعتبروها كارثة المجتمع.  العالم اضطرّوا يجو من بعد الأزمة عندن خجل، كان وضعن المادي منيح وفلّق صاروا مضطرين.  بفكّروا إنّو النوعية مش كويّسة، مش ولا بدّ، هات ايدك ولحقني، لا الحكما ولا الدوا ولا شي.  بفكّروا إنّو الأطبا يلّي بيجوا عالمركز مش شاطرين متل يلّي بالعيادات الخاصّة. | “As per our mentality, it is a care center for the poor, however the staff through their welcoming and conduct can alter this image, for sure” (UP, male, 71-79 age group)  “When I go to the hospital outpatient clinic, they welcome me warmly. When I went to the medical center, she made me feel indebted for the favor of providing services” (FMNU, male, 30-40 age group)  “I never go there when it is overcrowded, honestly, I can’t” (UP8, female, 71-79 age group)  “I am an old-women, I am sick and have body aches, I cannot go there and sit for three to four hours to get examined! This is constraining our ability to get medications, and we need these medications” (UP, female, ≥80 age group)  “My mom is an old-women, I cannot leave her alone for four to five hours while waiting to see the physician” (FMU, female, 51-65 age group)  “Once they see him at the wheel chairs, he doesn’t wait, even a minute” (FMU, female, 41-50 age group)  “It depends on one’s culture and information related to the funding of services” (NUP, male 71-79 age group).  “Older people are encouraged when they know the provider, and know how services are funded and why they are low- cost” (UP, female, 60-70 age group)  “There is a shared information that if you go to primary health care centers affiliated to the Ministry of Health, you won’t get a decent reception; Lebanese people consider that governmental or free services are below the required level” (NUP, male, 60-70 age group)  “Primary health care overcomes sectarianism” (NUP, male, 71-79 age group)  “Because it has this religious character, I don’t go” (NUP, male, ≥80 age group)  “Those are political centers, if you seek their service you will have to elect them” (FMNU, female, 41-50 age group)  “I personally do not like to seek care from a center with political affiliation, I feel I do not accept, I do not enter it, but I do not mind if it has a different religious affiliation” (FMNU, female, 41-50 age group)  “When I see these things, I hesitate, I might count to ten before I go and I say, what do I have to do there, they’re all strangers” (NUP, female, 60-70 age group).  “I prefer not going, I leave definitely, definitely” (UP, female, ≥80 age group)  “Lebanese are considered minority among foreigners in the center” (NUP, female, 71-79 age group).  “When I come to the center and I see this overcrowd and chaos, and non-Lebanese people are also seeking medications, it makes me feel that the center is not appropriate for me, I won’t come back” (UP, male, 71-79 age group)  “Sometimes they [non-Lebanese people] say we are treating you on our own account” (FMU, female, 51-65 age group)  “You can feel It [the attitude of some refugees indebting Lebanese people for services] through their eyes, their glances” (FMU, female, 30-40 age group)  “The center reputation motivates people to come, satisfied beneficiaries will send their peers, welcoming is being consistently great, it is not individual” (UP, female, 71-79 age group)  “It is according to reputation, what people are sharing” (FMNU, female, 51-65 age group)  “Those who are going are not finding their medications, so why should I go?!” (NUP, male, 71-79 age group)  “I know many people who think that PHCCs are for the poor, that’s why they don’t go” (NUP, male, ≥80 age group)  “Let people who have no insurance, pity for them, benefit of the center services.” (UP, female, 71-79 age group)  “Seeking care from a PHCC is perceived by the society members as a step-down” (FMNU, male, 30-40 age group)  “Society consider it [seeking care from a PHCC] as a disaster” (FMNU, female, 41-50 age group)  “They [people who became obliged to seek PHCCs services after the economic crisis] have shyness, they used to have a good economic status and now they are obliged to come to the center” (SP, more than 10 years of experience).  “A perception that the quality is low, not good, deficient. Neither physicians nor medications, nothing” (SP, more than 10 years of experience)  “Physicians who attend PHCCs are perceived to be less skillful than those who attend private clinics” (SP, up to 10 years of experience) |
| 2.2 Ability to seek | العامل الاقتصادي بحدد حركة البشر.  لعالم اكتر شي عم تتجاهل هيدا الشي من المادة. عآخر شي تا يروح.  شو هو اللي يمنعني أنا روح على الحكيم؟ هوي الوضع الاقتصادي. يعني إذا بدك تضطري تروحي بها لحالة بدك تكون مأمن المبلغ المعين إللي هو بدك تشوفي حكيم.  خيي توفى من سرطان القولون، المفروض إحنا الأخوة نعمل كل سنتين ناضور، هلق يمكن انا صرلي تلات سنين أو أربع سنين ما عملتو.  العامل المادي، بعد كل هالشي هوّي الدافع الأساسي للعالم ليروحوا ياخدوا خدمات من مراكز الرعاية.  قبل كنت اعمل فحوصات ادفغ عشرة بالمية او ما ادفع اليوم اذا بدي اعملن بكلفوا ١٠٠ دولار وانا معاشي كلو ٣٠ دولار  انا كنت كل سنة اعما تشك اب، كل سنة ، بطمن عن صحتي، بس هلأ لأ، حاليا لأ لأ لأنه ماني قادرة ماديا أعملهم.  قلي نسي الموضوع، في شيء أولويات أكثر ما بدي هلا ، انا ما بني شي، حاليا ما بدي  عايشين عحساب إبني، بستحي زيد عليه الحمل، بعتل همّو لأنوعندو عيلة.  أوّل شي صعب عليي تلفن، ما بعرف تلفن. تاني شي، بفكّر إنو ابني عندو ولاد وعيلة وجوزي بلا شغل، ليش تا شيل هالمبلغ من قدامن؟! يمكن بكرا إتحسّن.  بحسّ إنو عم بيزيد لحمل عإبنو.  ما عندن حدا يهتمّ فين، يرافقن، أو أهل بيتن موظفين، فبينطروا لحدّ ما يصير وضعم سيّء لحتى حدا من قرايبينن يجيبون، إذا عندن قرايبين.  بفضّل روح دغري عالمستشفى، أحسن ما إقعد جرّب إشيا جديدة.  العالم بتربط  بيستحيوا يروحوا ياخدو خدمات رخيصة أو بلاش.  توصيفن وفكرتن عن حكما الجامعة الامركيّة ممكن يتغيّر أذا بيعرفوهن إجوا على المركز.  العالم بيربطوا بين سعر المعاينة ونوعيتا.  يمكن اهلو وقرايبينو ما بيقبلوا ...يمكن انو ولادو ما بيكون عندن ثقة، او العالم بتحكي علين انن ما لهن قلب يروحوا عند حكيم اختصاصي وبدن يتحكموا عبلاش، العالم بيقولوا اخدوا اهلن عمراكز رخيصة.  بعدنا متجذرين بخلفيتنا الثقافية، مهما كان.  بتحسّ أغلب كبار السن بلبنان تعيسين، كئيبين وحاسين حالن تقال، ما بيرتاحوا يطلبوا مساعدة وبيتمنوا تكون حياتن قصيرة.  انو واحد يحب يتحكّم هيدا بدلّ إنو بحبّ الحياة وبحب حالو.  لمّا الإنسان بيكون بحاجة لدوا، بيروح وين ما كان بس ليحصل عليه. | “The economic factor determines human activity” (NUP, male, ≥80 age group)  “People are neglecting care mostly because of finances. They seek care at advanced stages” (UP, female, 60-70 age group)  “What do you think are barriers for me to go to the physician? It’s my financial status. Amidst this situation, one needs to ensure a certain amount before going to the physician” (UP, male, 60-70 age group)  “My brother passed away because of a colon cancer; we as brothers shall do a colonoscopy every two years, I haven’t done it since 4 years, I can’t afford it” (UP, male, 60-70 age group)  “The financial factor after all is currently the main driver for people to seek care from PHCCs” (NUP, female, 71-79 age group)  “I used to do my diagnostic tests and pay 10% or nothing. Today it costs hundred dollars and my whole salary worth thirty dollars” (UP, male, 60-70 age group)  “I used to do a yearly checkup, to get reassured about my health, but now no, currently no, no I can’t afford them” (UP, male, 71-79 age group)  “He told me: forget about it [a preventive test], I have other priorities, I am okay, I don’t want for the time being” (SP, up to 10 years of experience)  “We’re living on my son’s expenses; I feel ashamed to add to his burdens, I feel anxious about this because he has a family” (UP, female, 71-79 age group)  “First, it is difficult for me to make phone calls, I do know how to do. Second, I think that my children have families and my husband is unemployed, why to deprive them from this amount?! Maybe tomorrow I get better” (UP, female, 71-79 age group)  “He feels like he is adding a burden to his son” (SP, up to 10 years of experience)  “They do not have someone to care for them, to accompany them, or their family members are employees, so until the case becomes advanced so one of their family members bring them, if they have family members”. (SP, up to 10 years of experience)  “I prefer going directly to the hospital, instead of trying new settings” (FMNU, female, 41-50 age group)  “They feel ashamed to use cheap or free services” (UP, female, 71-79 age group)  “The description and perception about doctors who go to the American University Hospital would change if they came to the dispensary” (NUP, male, ≥80 age group)  “People correlate the value of the examination to its fees” (NUP, male, 60-70 age group)  “Maybe his children don't trust such services. People who gossip would say they do not dare to seek specialized services, they like free services, they took their parents to cheap centers” (FMNU, female, 41-50 age group)  “We’re still rooted in the cultural background no matter what” (NUP, male, ≥80 age group)  “You feel that most older people in Lebanon are so sad, they are depressed and feeling heavy, they do not feel comfortable asking for anything or seeking help, they would like to live shorter” (NUP, female, 71-79 age group)  “Seeking care to stay in good health is a form of loving life and one-self” (NUP, male, 60-70 age group)  “When a person needs medications, he goes anywhere just to get it” (NUP, female, 71-79 age group) |

Theme 3: Healthcare reaching

|  | Arabic (Lebanese) quotes | English translation |
| --- | --- | --- |
| 3.1 Availability and accommodation | أنا مثلا بجي من منطقة بعيدة، يمكن لو في حكيم أقرب هون كنت أنا ما جيت، ولكن أنا يلي باخدو بدل نقل ما باخد أجري بالمركز كمعاينة وهيدا بدل نقل معنوي أكتر منو مادي.  عملنا كتير تدريبات بالسنة يلّي مرقت بس ما كان خصّن بكبار السنّ.  جمعيّة الشبان المسيحيي عم تبعتلنا ربع الأدوية يلّي موجودين علايحة الأدوية تبعُن.  أغلب العالم بيحصلوا عدويان من أصل ثلاثة، ثلاثة من أصل خمسة، يعني. ما عم يشتروا يلّي ناقص لأنّو ما بيقدروا. يعني عم ياخدوا بسّ الدوا يلّي عم يلاقوا بالمركز، مش الوصفة كاملة.  ما لقيت غير شي دوايين من أصل عشرين، الرخاص بسّ.  الفيتامينات والمكملات الغذائية مش عم نلاقيها، بيعتبروها مش ضروريّة مع إنا موجودة عالوصفة، وبالفرمشيا غاليين كتير.  بالزيارات المنزلية منروح عند العالم يلّي عندن وضع ما بخلّيهن يجوا عالمركز، أو ما عندن حدا يجيبن.  بيجوا لعندي حكيم وممرضة وبيجيبولي الدوا كل شهر، لولاهن ما كان فيني روح، كيف بدي روح بحالتي.  يعني مثلاً إذا عندو وزن زايد، موجوع، أيّ حالو ما بيقدر ينطر، منمرّقو.  أيام بتروحي عمركز الرعاية بتلاقي عجق كتير، ٢٠ مريض عند هالحكيم بساعة، بقلك بفلّ، ما رح أوصل لنتيجة بهالعجقة، بفل.  المشكلة كمان بمراكز الرعاية الصحية الأولية هيي الاعداد. يعني اذا كان في خدمة عم تتقدم بكلفة قليلة عم بيكون في اقبال عليا بطريقة انو بيصير بدك تنطري لتحصلي عليا.  المراكز الصحية م عم يدفعوا إيام ما يعادل دولار واحد للمعاينة، يعني شي ٢٠ دولار ليشوف الحكيم ٢٠ مريض بالساعة.  لمّا بيدفعوا للحكيم عالساعة، هيدا الشي بيؤثّر على نوعيّة الكشفيّة لانّو الطبيب مضطرّ يسرع خصوصي لمّا عندو ضغط كتير.  المواصلات العامّة مش متوفّرة وتطلبي تاكسي غالي كتير، وتواحد يمشي عالمركز صعب خصوصي لمّا الطقس ما بيساعد.  مثل أنه مثلا إحنا بأمنولنا مختبر بالجيش فحص، بس تتنزلي، عالمختبر الأساسي بدك تحطي تنكة بنزين، يعني تنكة بنزين بدو يحط مليون وخمسمية الف، وهيو الفحص بكلف مليون.  مرضا السكّري أكيد بدّن مين يساعدن ليطلعوا الدرج ويوصلوا على عيادة العيون بالطابق التاني. | “I come from far away. If there was a physician who is closer, I would have not come. I consider what I am earning for my visits as a transportation reimbursement rather than honorarium” (SP, up to 10 years of experience)  “We attended many trainings last year, but none related to older people” (SP, up to 10 years of experience)  “The YMCA is supplying only 25% of their predetermined list of medications” (SP, up to 10 years of experience)  “Most people are getting two medications out of three, three out of five. They do not buy the remaining, they cannot afford. They are taking only the medications that are provided here, not the full prescription” (SP, up to 10 years of experience).  “Two out of twenty medications were only available, the cheapest ones” (UP, male, ≥80 age group)  “Food supplements are not provided and not considered as essential even if prescribed, they are expensive at pharmacies” (UP, female, 60-70 age group)  “Home visits target people with critical cases, those who cannot move to attend the center, or have no one to accompany them” (SP, up to 10 years of experience)  “A physician and nurse visit me monthly and bring my medications, otherwise I wouldn’t be able to get care, I cannot go to the center in my case!” (UP, female, 71-79 age group)  “If he’s for example obese, in pain, presents any case that constrain him from waiting, we would let him pass” (SP, up to 10 years of experience)  “Sometimes when you go to the center and you find this overcrowd, 20 patients to be examined in one hour, you would leave, you cannot get good results amidst this overcrowd” (UP, female, ≥80 age group)  “The problem with health care centers is the high census; there is a high demand on low-cost services which increases the waiting time” (NUP, male, 60-70 age group)  “PHCCs do not commit to the tariff fixed by the order of physicians for the medical consultation it could be 1$ per patient resulting in 20$ for 20 patients per hour” (SP, more than 10 years of experience).  “Paying the physician per patient may affect the quality of care as physicians could speed up the examination to see more patients per hour, especially when need is high” (SP, more than 10 years of experience).  “Public transportation is not available, ordering taxi is expensive, and it is hard to walk to the PHCC especially in extreme weather” (UP, female, 60-70 age group)  “We [retired soldiers] are eligible to do lab-tests at the army laboratory, but to get there you need a gasoline gallon that costs one million five hundred thousand, which exceed the cost of the test itself” (UP, male, 71-79 age group)  “Diabetic patients need help, indeed, to take the stairs and reach the ophthalmologic clinic at the second floor” (FMU, female, 51-65 age group) |
| 3.2 Ability to reach | كبار السن يلي بعانوا من صعوبات بالمشي بدّن مساعدة ليوصلوا عالمركز، خصوصي إذا جايين لوحدن. إيام بينطروا لحتى يفضا كرسي متحرّك.  بسبب عمرن، كبار السن بدّن مساعدة، مين يدلّن، يسوق فيُن. | " OP with physical limitations need assistance to get into the center if they come alone. They need to wait sometimes to get a free wheel chair" (SP, up to 10 years of experience)  “Older people need assistance, someone to guide, to drive, because of their age” (FMNU, female, 41-50 age group) |

Theme 4: Healthcare utilization

|  | Arabic (Lebanese) quotes | English translation |
| --- | --- | --- |
| 4.1 Affordability | بالعادة كان يوصلنا دعم كبير للبرامج، هلّق ما بقى يطلع شي.  بالأوّل كان مفتوح، مبالغ مفتوحة، كنّا نقدر نغطّي كلّ العالم يلي تجي، قدّ ما بدنا معاينات، ما في مشكلة. بعد حرب أوكرانيا، صارت المبالغ محدودة، كتير قلّت وبلّغونا أنو بدّا تصير أقلّ بعد.  الحاجة بمنطقتنا بتفوق الدعم يل] عم نحصل عليه.  كان عنا ١٤ مريض بجمعية الشبان المسيحية، بتعرفي كم مريض هلق؟ ١٤٠٠ مريض.  العالم ما عادت قادرة تدفع التكاليف بالعيادات الخاصة، لا معاينات ولا فحوصات.  انا بالأوّل بسأل عن يلّي بيشتغلوا بالمركز، الكلفة ما بتهمّ، ما بتفرق معي إذا الخدمات رخيصة.  أنل شخصيّاً بتفزعني الخدمة يلّي سعرا قليل، بحسّا مش منيحة، وإذا عندي القدرة ما بختارا. | “We used to get large funds, now it’s almost nothing” (SP, up to 10 years of experience).  “At the beginning it was open, open budget, we were able to serve as many people as we get, medical examinations as needed, no problem. After the Ukrainian war, they limited the fund. It has shrunk, and they informed us that it will decrease more” (SP, up to 10 years of experience)  “The need in this region is exceeding the support we are getting” (SP, up to 10 years of experience)  “We used to have 14 subscribers on the YMCA program; do you know how much we got after the crisis? 1400 subscribers” (SP, up to 10 years of experience)  “People are not able anymore to afford services provided at private clinics, neither the cost of medical consultations not the cost of diagnostic tests” (FMU, female, 51-65 age group)  “I firstly seek information about the operating staff before choosing the medical setting, cost is not important, I don’t mind low-cost services!” (FMNU, female, 51-65 age group)  “I personally fear low-cost services, I see them as deficient, if I am able, I won’t choose them” (FMNU, female, 41-50 age group) |
| 4.2 Ability to pay | إذا كان ماديا مرتاح، أكيد ما رح يروح ينطر مثلا ٣ او ٤ ساعات بالمستوصفات تكان ينطر، تكان يتحكم، بفضل إنه يفوت عالمستشفى ويدفع من ماله، إذا كان قادر؟  الكبار بالعمر عم يتحمّلوا كتير بهالايام.  عم نلاقي كتير صعوبات بما يخصّ العلاجات والأدوية، العالم ما معا مصاري ومش قادرة تشتري دوا.  معاشك هوي بأثر عليكي، أنا ما بدي روح بس مضطر صرت.  قبل كنت اعمل فحوصات ادفغ عشرة بالمية او ما ادفع اليوم اذا بدي اعملن بكلفوا ١٠٠ دولار وانا معاشي كلو ٣٠ دولار.  هلّق أنا مضمون عحساب الضمان الإجتماعي، بس ما فيني إتحكم إذا ما في بجيبتي مصاري.  حتى ولو قدّمت ليردولك من الضمان، بدك تنطر سنين ليرجعولك مصرياتك. فنفس الشي إذا عندك أو ما عندك ضمان. بهالحالة واحد بيرم وين في مركز رعاية صحيّة حتى يحصل عالخدمات.  ايه منخاف، لا سمح الله اذا صار عليي عارض صحي مين بدو يتحملني؟ عندي خوف اكيد.  عايش كبير السنّ بهاجس انو انا اذا صابنب شي شو بدي اعمل؟! بتضل نفسيتو تعبانة؟  غياب ضمان الشيخوخة اهم شي، الدولة مانا موجودة.  إذا واحد ما عندو دعم من برّا ما بيقدر يعيش ويكفّي، ولا ممكن. هيدي خبرتي أنا.  انضربت الطبقات كلا ، كل الطبقات صارت فقيرة. نحنا كبار السن عايشين عالة على ولادنا، او عأرضنا او عبيتنا، ما بق عنا شي.  أخذت الفواتير وضليت اكتر من ساعة ونص ناطرة قدام البنك متل الشحادة ، ساعتين ونص قدام البنك لقبضت قرشين من الفاتورة.  حتى يلّي كان معو بالبنك ما بيقدّوا مع غلا هالمعيشة والتضخم يلّي صاير مع الازمة، ما بقى يسوا شي، وواحد ما في يكفّي بلا دعم من حدا من أهليتو عايش برا.  المدخول تبع اليوم ما عاد يقدي لنروح عند حكيم. عندك كتير فواتير والاكل، عم نبدي شي على شي لأنه المصريات بالبنك راحوا .  بسبب الضيقة الاقتصادية قلّت الحكمة، الا يلي عم بيموت ما عم بيروح عالمستشفى. | “Well-off people won’t go wait for three to four hours at the primary care center to get the service. They seek care from the hospital outpatient clinic and pay as long as they can afford the cost” (UP, female, 60-70 age group)  “The older person bears as much as he can these days” (UP, female, 71-79 age group)  “I face a major difficulty related to treatments and medications; people do not have money and cannot afford their medications” (SP, up to 10 years of experience)  “Your income affects your behaviors. I don’t want to go to primary care centers but I am obliged” (FMNU, male, 30-40 age group)  “I used to do my diagnostic tests and pay 10% or nothing. Today it costs hundred dollars and my whole salary worth thirty dollars” (UP, male, 60-70 age group)  “I am insured with the National Social Security Fund, but cannot get care if I do not have money” (NUP, female, 60-70 age group)  “Even if you submit for reimbursement you wait for years to get your money back, having and not having this type of insurance is the same. In this case you search for primary care centers to seek care from” (NUP, female, 71-79 age group)  “Yes, we are anxious, if any health issue happens to me, who will stand by me? definitely I am afraid” (UP, male, 60-70 age group)  “The older person lives with an obsessive idea, a constant mental distress: what do I do if any health problem happens to me?!” (UP, male, 71-79 age group)  “The lack of old-age insurance is essential. The government is absent!” (NUP, male, 71-79 age group)  “If one’s is lacking external support, he cannot live and sustain, at all. This is my personal experience” (NUP, male, 71-79 age group)  “Social classes are abolished; all people became poor. As older people, we became a burden for our families, homes, and country, we have nothing anymore” (UP, male, 60-70 age group)  “I took the invoice and waited for more than an hour and a half in front of the bank, like a beggar, to get reimbursed. Two hours in front of the bank to collect pennies from the invoice” (UP, female, 60-70 age group)  “Even huge savings are drained due to the current situation and inflation, it worth nothing, and cannot sustain without the support of family members who live abroad” (NUP, male, 71-79 age group)  “Today’s income is not enough even to get a medical examination. One has multiple bills; we are resetting our priorities because our savings collapsed at banks” (FMNU, male, 51-65 age group)  “Because of the economic crisis, people are seeking care less, only people who are dying are going to hospitals” (NUP, male, 71-79 age group) |

Theme 5: Healthcare consequences

|  | Arabic (Lebanese) quotes | English translation |
| --- | --- | --- |
| 5.1 Appropriateness | كانك إنتي بعيادة وعم فوتي عند حكيم تحضرك الممرضة وتفوتك عند الحكيم والحكيم حرام عم ياخد وقته.  كلا سؤالات حكي بحكي إنه تا يشوفو النبض او يحطوا سماعة عرواياكي او عقلبك ما في.  منحكي وبس... بدّن الصرفة تفلّي حتى غيرك يفوت، صارت معي، كويّس وكلّ شي بس كلّو عالسريع. بدّن يركضوا ويلحقوا عكذا مريض هون وهونيك.  ولك بدّك تسألين عن حالك، عن جسمك، ما فيكي، كلّن مستعجلين.  هوي وعم يكتب الدوا بيحكي. ما بيطلع فيكي.  لما بالساعة بحطلك 30 أو 40 مريض ماعندك وقت تطلعي بعيون المريض.  ما بيعطي وقت الحكيم بالمركز متل بعيادتو. يمكن لأنو في ضغط كتير، بدو يساعد أكبر عدد ممكن.  كان برا ناطر شي ٢٠ مريض، فوتني ٣ ثواني وقللي رجعي مرقي لعندي عالعيادة.  بمراكز الرعاية، يعني اللازم، إنتي عندك ضغط بزنلك ضغطك بيعطيكي دوا ما في أمتين بلّش معك وكيف؟ ما في وقت هيدا عامل الوقت، يعني الحكيم كتير مضغوط، لأن نسبة العالم إللي عنده وهوي بيجي مرة بالجمعة، عرفتي، ما عنده وقت أكيد.  لا، لا، لا، ابداّ ما دقرني، قلي رح نعمل هلّق فحص دم وبفحصك بعدو. وما عطيوني خبر ايمتى وكيف فيني إحصل عالنتايج.  فحصني الحكيم، وصفلي الأدوية وهنّي أمّنولي ياهن.  بركّزوا أكتر شي عالسبب يلي انتي جايي كرمالو.  الأسئلة متعلقة أكتر بالحالة يلي هو فيها.  مشاكل الذاكرة مش إختصاصو.  هلق إنه بيشرح بس إنه مش ها الشرح التفصيلي، يعني بيجي بيقعد يدققلك بتفاصيل.  أنا بدي أدرس يعني نص ربع حكيم لحتى كمان أنا أكون فاهمة الخبرية لحتى أقدر اشرحلو.  ما حدا، ما حدا بيسأل عن هالإشيا. ما حدا بيسأل إلا إذا جايي كرمالن.  بصراحة ولا مرة صادفت طبيب توسع بالامور هيدي، دايما بركز عمرضك وبيوصفلك العلاج، انما ما بيفوت معك بتفاصيل اذا حدا بيهتم فيك، حياتك كيفيي، شو عم تحس، نفسيا كيف عم تتعامل مع الاحداث، مع مرضك، قليل، ما في هالثقافة كتير بلبنان  لا، لا، لا، لا ما. ما الو علاقة بي هاي الشغلة، هو بيسألوا عن القلب، قديش صرلك ماانك عامل فحوصات، بدي يكتب له تا يروح يعمل فحوصات يللي مختص فيه، لا ما بفوت بشي ثاني.  القصّة أنّو الأطباء منن بوارد يعملوا شغل زيادة. يمكن بيقولوا عم يطلعلي دولار او اثنين عكلّ مريض، ما حإجي دخّل معلومات فوق هيدا كلّو، ما بيقبلو.  كان عنّا مشروع مموّل من الأي ام سي لدعم كبار السن... هيدا كان كتير منيح، بس توقّف هلّق.  أي اختصاص فايتي لعنده بيغوص بمجاله، مثلاً حكيمي نسائية ممكن تتطرق لصور وهيك شي، بس اذا الطب العام ما بيسأله للمدام إذا عاملي فحص سرطان عنق الرحم.  ما بتعودي تفكّري بحالك إلّا يالإشيا الضرورية. مثلاً انا نطرت لحدّ ما عدت شفت بعيوني لحتى رحت عند الحكيم.  لحتّى تعملي شي لازم يكون ضروري، وإلا ما بتعمليه.  هودي ما عندي ياهن بقى، عاملتهم، أنا وقت إللي كنت، اولد من هيك  بقلك لاء، لانو بدو يفكر انو بدو يحط مصاري، ما معو، ليكي انا رح ابكي، انو عايشين عحساب ولادنا ، ولادنا عم يبعتولنا تناكل، عم يشتغلوا ويتعبوا بلا ما حطا هون بجيب فيها كيلو لحمة  انا كنت كل سنة اعمل تشك اب، كل سنة ، بتطمن عن صحتي، بس هلأ لأ، حاليا لأ لأ لأنه ماني قادرة ماديا أعملهم.  بالأوضاع يلي مارق فيها البلد في مرات واحد باجا اشيا اذا مش كتير ضرورية بسبب كلفتا العالية.  حسب خبرتي، بلبنان كل العلاجات يلي عم تصير هيي علاجات بسبب إحساس مرض معين، الاشيا الوقائية ما عنا ثقافة فيا.  أنا عن روح جيب أدوية، أيش بدي بغير شيء أنا ؟  إنك ترتاح للطبيب هيدا شي أساسي لتخبرو كلّ شي. إذا مش مرتاح معو ما بتخبرو شي.  حسب الطبيب، الثقة، وأخلاقو.  بتعرفي انو قصص حياتن ياما باكيين معن نحنا، ياما مخبرينا يلي ما بيتخبر حتى لولادن.  اللياقة والاحترام موجود بس في ضغط مرضى بشكل هالمشكلة.  إللي عم لاحظه إنه أنت بدك تعرفي أنت شو حالتك، وأنت بدك تحكي مع الحكيم وتقوليلو . يعني أنت بدك تجبريه يأخذ وقته معك وتخبريه.  إيه بالخاص إيه، إيه بيعطيكي الوقت الحكيم بتنزلي عالوقت بتفوتي على الوقت، بياخد وقته معه وبيحكي معه وبيمزح معه، ، بينما يمكن إذا بأخذه مستوصف لا ما نو فاضيلو.  متل كأنن عم بمرّقوا أرقام بسرعة يلا، كيف ما كان، بلا إهتمام.  وبطبشلك عضهرك وخلص. يا تانت منيح صحتك بتجنن/ ما بكي شي،". صار عمرك هالقد منيح انشالله نصير قدك قواي امتلك. | “As in private clinics, a nurse prepares you and accompany you to the physician who dedicates appropriate time” (FMU, female, 51-65 age group)  “Consisting only of questions, just talking with no attempts to measure your pulse or put a stethoscope to check your lungs and heart” (UP, male, 71-79 age group)  “We just talk… They want you to leave so that someone else comes in. It happened to me, he is good, but it’s all fast, fast. They are running out of time, and they need to handle many cases, at many settings” (UP, female, 71-79 age group)  “You need to ask them about your body, but you can’t. they’re in rush” (NUP, female, 71-79 age group)  “He talks to me while writing, he does not look at my eyes” (UP, female, 71-79 age group)  “When they assign me thirty or forty patients per hour, I cannot even look at their eyes” SP, physician, more than 10 years of experience)  “The physician doesn’t allocate as much time as he does at his clinic. Maybe because of the overcrowd, he wants to serve as many as possible” (NUP, female, 60-70 age group)  “There were almost twenty patients waiting for him in the outside, I walked in for three seconds and he advised me to go to his clinic” (FMNU, female, 41-50 age group)  “At primary care centers they do what is strictly necessary; if you present high blood pressure, they measure it and prescribe medications. Questions about since when it happened and how it happened do not exist. There is no time, the physician is extremely overwhelmed because he attends the center once a week and there is a high demand, you know, he does not have time, indeed!” (FMU, female, 51-65 age group)  “No, no no, no, he didn’t touch me. They said we will do now a blood test then I will examine you later on. They did not even tell me how and when to get the results” (UP, male, ≥80 age group)  “The physician examined me, prescribed medications and they brought them to me” (UP, female, 71-79 age group)  “They focus only on your chief complaint” (NUP, male, 71-79 age group)  “Asked questions are centered on the presented case” (SP, 5 years of experience)  “Memory problems do not fall under his specialty” (NUP, male, 71-79 age group)  “He explains but not that extensively” (FMNU, male, 41-50 age group)  “I need to be a half doctor to understand the case and explain it to the physician” (FMU, female, 41-50 age group)  “No, no one ask about these. No one ask, if you do not present it or not coming because of it, no one will ask” (UP, female, 71-79 age group).  “Honestly, I have never come across a doctor who elaborates on these matters. He always focuses on your disease and prescribes treatment, but he doesn’t go into detail such if you have a familial support, your life, how you feel, how you deal psychologically with events and your illness. It rarely happens. We lack this culture in Lebanon”. (UP, male, 71-79 age group)    “No no no, he has nothing to do with these questions, he asks about heart problems, heart checkup, he prescribes related tests but does not interfere in anything else” (FMNU, female, 41-50 age group)  “The issue is that physicians do not want to do additional work. They may say we are earning one or two dollars for each patient, so we won’t manage data on top of this, they do not accept” (SP, up to 10 years of experience)  “We have a project funded by IMC (NGO) to support people who are aged … this was a great project, but it is being stopped now” (SP, up to 10 years of experience)  “Every specialized physician will delve into his field. For example, a gynecologist might discuss mammograms and such things. But if he is a generalist, he won’t ask the lady if she has done a test for cervical cancer”. (FMU, female, 51-65 age group)  “You no longer think about yourself except about what is necessary. For example, I waited to the point that I was not able to see with my eyes anymore, that I went to the physician” (UP, female, 71-79 age group)  “To do something it has to be urgent, otherwise you do not” (UP, male, 60-70 age group)  “I don’t have to do these tests [preventive tests] anymore, I used to do them when I was younger” (UP, female, ≥80 age group)  “No, older people are not doing such tests [preventive tests], one would think about their cost, look I am about to cry, we are living on our children expenses, they are sending us money to eat, they work hard to send us money, instead of paying for these unnecessary tests I buy one kilo of meat” (UP, female, age 67)  “I used to do a yearly checkup, to get reassured about my health, but not anymore, currently no, no I can’t afford” (UP, female, 60-70 age group)  “In view of the current situation at the country, one delays things if not urgent, because of their high cost” (NUP, male, 71-79 age group)  “Based on my experience, disease is the main driver to get care, we don’t have the culture of preventive care” (NUP, male, 60-70 age group)  “I’m going there [to the PHCC] and getting my medications, this is all what I care for” (UP, male, 71-79 age group)  “Getting on well with the care provider is important to tell him everything. If you don’t feel comfortable, you won’t say anything” (NUP, male, 60-70 age group).  “This depends on the physician, trust, morals, values” (NUP, female, 71-79 age group)  “We heard their stories, we cried with them so many times, they tell us what they do not disclose even to their children” (SP, up to 10 years of experience)  “Courtesy and respect are present but there is much pressure because of the overcrowd, this is the problem” (UP, female, 71-79 age group)  “I am noticing that you need to get some awareness about your case, to be able to discuss with the physician, so you force him to provide time for discussion” (FMNU, male, 41-50 age group)  “At private clinics you go on time, get examined on time, the physician provides enough time to talk to him, joke with him, however at the medical center, no, the physician does not have much time for him” (FMNU, female, 41-50 age group)  “It is more like “passing numbers quickly, no matter how, without special attention” (NUP, female, 71-79 age group).  “He taps on your shoulder and that’s it. He says: you’re in good health mam, you have nothing. You should be thankful you’ve got to this age; I hope we age like you and stay that strong!” (UP, female, 71-79 age group) |
| 5.2 Ability to engage | بخجل إسأل، لأني مش متعلّمة.  لكن انا بعرف متل الحكيم! متل ما بيقلي بعمل.  هوّي لو بيعرف مزبوط شو الحالو وشو صاير معو كان اكيد بيقدر يناقش أكتر بخطّة العلاج.  في ناس عندن ضعف بالسمع، يمكن بيقولوا حتى ولو سألت ما رح إقدر إسمع الجواب. فما بيسألوا.  حتى استقبال الحكيم كيف بيأثر كثير على الكبار أو على الصغار.  إذا المريض مرتاح للطبيب بيسأل، بس إذا بحسّ إنّو الطبيب أنجأ بيجاوبو وبردّ عليه بنشافة وما بيفتحلو مواضيع، ساعتها بيحسّ إنّو لازم يوقف يسأل ما بيعود يناقش.  عصّب، مسك الروشيتا والقلم وقلي تفضل قيّد انتي!  ما شي بيمنعني ناقش/ بسّ إذا الحكيم بيسمعلي.  في حكما ما بترد، انو حكمتك فحصتك عطيتك الدوا وروحي عبيتك. اذا برد اكيد منناقش.  هني بحسسوا المريض انو سآل بعد، من طريقتو كيف بيحكي مع المريض وبيقلا مثلا شو كمان؟ شو بتحسي؟ يعني بيحكي معا. أوقات بتحسي ناشف ، أنجأ يجاوبا ما بيطلع فيها ، ما بيعود الا عين تسأل سؤال ثاني، كيف هوي عم يتعامل معا يا بيفتحلها مجال يا إما بتحسو ناشف ما بيعود الا عين تسألو.  إنتي لازم تعرفي شو حالته، وإنتي بدك تقولي له أنا بدي أكون فاهمة الخبرية لحتى أقدر اشرحلو  إذا حكيم مضغوط بيكون عندو مشكلة. | “I feel shy to ask because I am not educated” (UP, female, 71-79 age group).  “Do I know as much as the physician?! I do whatever he says” (UP, female, 71-79 age group)  “If he really knows what’s happening to him, he could better engage in the care plan” (NUP, female, 60-70 age group).  “Some people have hearing limitations, they would say, even if I ask, I won’t be able to hear the answer, so they do not ask” (SP, up to 10 years of experience).  “The physician’s welcoming affects adults and children likewise” (FMNU, female, 41-50 age group)  “If the patient feels comfortable with the physician, he will ask, but when he feels that the physician is providing strict and short answers and not tackling further issues, he feels the need to stop engaging, he won’t continue” (NUP, female, 60-70 age group)  “He got mad [the physician], he gave me the prescription paper and pen and told me: you prescribe!” (UP, male, 71-79 age group)  “Nothing matters, but only if he [physician] listens to me” (UP, female, 71-79 age group)  “Some physicians do not answer… if they answer we discuss” (UP, female, 71-79 age group)  “The way they talk may encourage patients to ask; like saying what else? What do you feel? Sometimes you feel the physician is dry, he barely answers, does not look at her, she won’t dare to ask further” (FMNU, female, 41-50 age group).  “The family member needs to know the case to explain to the physician” (FMNU, female, 41-50 age group)  “It gets problematic when the physician is overwhelmed” (FMNU, Male, age 51-65 age group) |
