# Supplementary material for: Barriers and enablers to primary health care center access for older people in Lebanon: A qualitative inquiry
Source: PLoS One. 2025 Oct 23;20(10):e0335073. doi: 10.1371/journal.pone.0335073 (PMC12548930; doi:10.1371/journal.pone.0335073)
Supplement: S5 File — (DOCX) [file pone.0335073.s005.docx]

| **Supplementary File S5. Code classification into categories, subthemes, and themes** | | | |
| --- | --- | --- | --- |
| **Themes** | **Subthemes** | **Categories** | **Codes** |
| Perception of needs and desire for care | Approachability | Information on available services | Lack of knowledge on PHC  Lack of knowledge on PHCCs  Mixing between PHCCs and dispensaries |
|  |  | Source of information | Informal channels  Formal channels |
|  | Ability to perceive | Socio-demographic determinants | Education and health literacy  Age  Living arrangement  Socioeconomic status |
|  |  | Health determinants | Health needs  Emotional status  Cognitive abilities |
|  |  | Service-related determinants | Free services |
| Healthcare seeking | Acceptability | Provider-related determinants | Staff behavior and attitude  Provider’s characteristics  Negative role of physicians |
|  |  | Service-related determinants | Service organization  Availability and quality of services  Information about services |
|  |  | Cultural and social determinants | Trust in governmental services  Cultural aspect of the center  Influx on non-Lebanese beneficiaries  Shared experiences  Critical negative perception |
|  | Ability to seek | Socio-demographic determinants | Socioeconomic status  Gender  Education and health literacy |
|  |  | Social and cultural determinants | Family and social support  Roles and functions  Religion  Personal beliefs and attitudes |
|  |  | health determinants | Emotional status  Physical abilities  Health needs |
|  |  | Environmental determinants | Travel distance  Transportation |
| Healthcare reaching | Availability and accommodation | Service-related determinants | Availability of professionals  Availability of medications and equipment  Scope of services  Service organization |
|  |  | Environmental determinants | Travel and transportation  Built environment |
|  | Ability to reach | Socio-demographic determinants | Age  Dwelling areas |
|  |  | Health determinants | Mobility and assistive devices |
| Healthcare utilization | Affordability | Economic determinants | Availability of geriatric funds  Transportation cost |
|  |  | Service-related determinants | Service fees  Contribution fees |
|  | Ability to pay | Socio-demographic determinants | Socioeconomic status  Insurance and pension plans  Familial and social support |
|  |  | Economic Determinants | The economic crisis |
| Healthcare consequences | Appropriateness | Service-related determinants | Geriatric clinical examination  Care coordination  Care continuity |
|  |  | Provider-related determinants | Care comprehensiveness  Client-provider relationship |
|  | Ability to engage | Socio-demographic determinants | Education and health literacyTrust Self-efficacy and interest |
|  |  | Health determinants | Cognitive and sensory abilities Emotional status |
|  |  | Provider-related determinants | Providers’ conduct Providers’ communication skills |
|  |  | Social and cultural determinants | Role of family members Alternative source of information |
|  |  | Service-related determinants | Examination time and fees |
