# Supplementary material for: Barriers and enablers to primary health care center access for older people in Lebanon: A qualitative inquiry
Source: PLoS One. 2025 Oct 23;20(10):e0335073. doi: 10.1371/journal.pone.0335073 (PMC12548930; doi:10.1371/journal.pone.0335073)
Supplement: S6 File — (DOCX) [file pone.0335073.s006.docx]

**Supplementary File S6. Mapped summary of access barriers and enablers**

| **Factors per service dimensions and older people’s abilities** | **Barriers** | **Enablers** |
| --- | --- | --- |
| **Approachability** | | |
| **Information on available services** | Lack of knowledge on the PHC concept and PHCCs services, locations, eligibility criteria, funding sources. | Available information about providers. |
| **Source of information** | Informal source of information (word of mouth). | Outreach activities.  Active role of OP, staff members and municipalities in spreading information. |
| **Ability to perceive** | | |
| **Socio-demographic determinants** | Living alone (carelessness). | Education and health literacy.  Previous lived experience.  Age.  Living alone (adverse health outcomes requiring care).  Familial support. |
| **Health determinants** | Fear from disease and death.  Limited cognitive abilities.  Ability to bear symptoms.  Health need (mental, functional and physiologic issues).  Stress, sadness, and fear (carelessness). | Stress, sadness, and fear (health concerns and adverse health outcomes requiring care). |
| **Service-related determinants** |  | Free services and campaigns |
| **Acceptability** | | |
| **Provider-related determinants** | Staff negative behaviour and attitude:  - Unprofessional, rude, and careless conduct;  - Poor communication skills (yelling and talking with superiority);  - Favoritism, politicism.  Negative role of physicians who discourage the use of PHCCs. | Staff positive behaviour and attitude:  - Warm and respectful welcoming;  - Positive communication skills (compassion words, active listening);  - Treating people equally and respecting the queue.  Positive client-provider relationship.  Being able to choose the provider.  Providers characteristics: specialized, skilful, experienced with humane and ethical conduct. |
| **Service-related determinants** | Service organization:  - Long waiting time;  - Overcrowd;  - Lining-up;  - Delayed physician’s arrivals.  Lack of information about the funding of low-cost services and the non-Lebanese supportive programs. | Service organization:  - Prioritization of older people with disability and critical cases.  Availability and quality of services:  - Availability of chronic medications;  - Availability of skilful providers. |
| **Cultural and social determinants** | Lack of trust in public services in general and generic medications.  Political or religious clear affiliation of the PHCC.  Massive influx of Syrian displaced people.  Prioritization of non-Lebanese people.  Shared negative experiences.  Critical negative perception:  - Stigma: labelling PHCC beneficiaries as poor;  - Shared negative perception regarding the quality of services;  - Perception among non-users that using PHCCs deprive disadvantaged people from their rights. | Trust of services delivered through PHCCs.  Leadership and management at the centre level.  Shared positive experiences. |
| **Ability to seek** | | |
| **Socio-demographic determinants** | Poor socio-economic status (lack of access even to PHCC services).  High educational level (choice of private settings).  Living in overpopulated areas (high demand overcrowding PHCCs). | Limited financial abilities (choice of PHCCs).  Economic crisis.  Education and health literacy.  Gender: being a woman. |
| **Social and cultural determinants** | Living alone (incapacity/neglect).  Having a role or a duty (limited time availability).  Religious beliefs (healing beliefs and attitudes toward PHCCs led by religious entities).  Political beliefs (in relation to PHCCs led by political entities).  Personal attitudes:  - Saving available resources for the most disadvantaged;  - Adherence to the same provider;  - Lack of trust in public services;  - Perception of low-quality services at PHCCs. | Family and social support.  Having financial responsibilities.  Attitude of taking advantage of free services. |
| **Health determinants** | Fear of contamination and medical procedures.  Depression.  Lack of autonomy:  - Cognitive limitations;  - Physical dependency;  - Financial dependency. | Fear of sickness.  Successful ageing.  Need for medications.  Low-acuity health conditions. |
| **Environmental determinants** | Long travel distance.  Unavailability of transportation.  High transportation cost. |  |
| **Availability and accommodation** | | |
| **Service-related determinants** | Availability of professionals:  - Physicians’ restricted attendance at PHCCs;  - Absence of a general practitioner during all opening hours;  - Shortage of physicians;  - Lack of specific geriatric training.  Availability of medications and equipment:  - Chronic medications shortage;  - Lack of advanced equipment;  - Lack of assistive devices.  Scope of services:  - Lack/discontinuation of specific geriatric services and programs;  - Lack of specialized care, diagnostic tests, screening tests, daily blood testing, health education, and vaccines for older people.  Service organization:  - Restricted opening hours (unsuitable for family members of OP);  - Overcrowd;  - Long waiting time (up to 4 hours);  - High census of patients per hour of examination;  - Inappropriate conservation and packaging of medications;  - Unfair payment for physicians;  - Mal-organized services – favouritism. | Availability of professionals:  - Presence of skilful physicians from all specialties;  - Appropriate staffing.  Scope of services:  - Homecare services.  Service organization:  - Possibility to call and make appointments;  - Organized appointments;  - Comfortable waiting rooms equipped with TVs;  - Short waiting time;  - Limited number of patients per hour of examination;  - Fair payment to physicians;  - Availability of personnel to assist the walk-in of older people with disability;  - Reminder calls to pick-up medications;  - Outreach activities to promote preventive tests;  - Computerized records;  - Prioritizing OP. |
| **Environmental determinants** | Lack of common transportation.  Expensive transportation cost.  Absence of lifts. | Short travel distance-proximity.  Presence of lifts and ramps. |
| **Ability to reach** | | |
| **Socio-demographic determinants** | Advanced age. | Urban dwelling area (transportation infrastructure). |
| **Health determinants** | Limited mobility or physical disability.  Lack of assistive devices like wheel chairs. |  |
| **Affordability** | | |
| **Economic determinants** | Availability of geriatric funds.  - Lack of support from municipalities (during the economic crisis).  - Decreased funds provided by external agencies.  Increased transportation cost. |  |
| **Service-related determinants** | Low-cost services (linked to low-quality). | Service fees:  - Low-cost services;  - Expensive services at private settings;  - Specific consideration and reductions for people with disability or with financial limitations. |
| **Ability to pay** | | |
| **Socio-demographic determinants** | Good financial status (choice of private providers).  Having insurance (choice of private providers).  Lack of old-age insurance and pension plans. | Limited financial abilities (choice of PHCCs).  Familial and social financial support. |
| **Economic determinants** | Inflation and high living expenses. | Dysfunctional public insurances.  Currency devaluation.  Bank-system failure. |
| **Appropriateness** | | |
| **Service-related determinants** | Geriatric clinical examination:  - Short duration;  - Shallow examination consisting mainly of talking;  - Inconsistent application of patient preparation across centres;  - Examination focused on the chief complaint;  - Fragmented care (each specialist within his scope);  - Restricted explanation;  - Uncomprehensive and unstructured geriatric assessment.  Care coordination:  - Lack of individual computerized record across settings;  - Lack of referral systems;  - Providers’ inability to check the record details;  - Physicians not filling-in computerized records.  Care continuity  - Medication shortage;  - Discontinuation of supportive programs. | Geriatric clinical examination:  - Nurses’ qualification and contribution to the examination.  Care coordination  - Role of family members in providing comprehensive information;  - Computerized files at PHCCs;  - Role of nurses in filling-in computerized records.  Care continuity:  - Being examined by the same physician over visits. |
| **Provider-related determinants** | Client-provider relationship:  - Careless attitude;  - Poor communication skills during the examination;  - Lack of time to interact and set a pleasant environment;  - Paternalistic approach;  - Materialistic approach;  - Disinterest regarding the social and financial aspects of beneficiaries;  - Under-estimation of OP’s complaints;  Negative communication:  - Yelling;  - Getting angry of people who ask;  - Not maintaining eye contact;  - Rude verbal statements;  - Using technical terms to explain;  - Lack of explanation.  Care comprehensiveness:  - Therapeutic oriented care;  - Lack of clients’ interest in preventive care;  - Lack of health education. | Client-provider relationship:  - Trustful client-provider relationship;  - Users coming prepared with some awareness about the case.  Positive communication:  - Providing explanation in plain language;  - Delivering quality care irrespective of the setting type;  - Caring for OP’s concerns;  - Asking questions;  - Listening actively;  - Providing counseling;  - Calling to follow-up;  - Manifesting cultural competence. |
| **Ability to engage** | | |
| **Socio-demographic determinants** | Fearing physicians.  Over-trusting physicians. | Education and health literacy.  Fearing sickness.  Self-efficacy and curiosity. |
| **Health determinants** | Sadness, depression, and hopelessness.  Cognitive and sensory limitations. | Preserved cognitive abilities. |
| **Provider-related determinants** | Negative communication skills:  - Getting rude over OP who argue;  - Not explaining or saying what is strictly necessary;  - Serious facial expressions;  - Talking while writing. | Positive client-provider relationship:  - Warm welcoming;  - Caring for their concerns;  - Asking about them and following up.  Positive communication skills:  - Asking questions;  - Listening actively;  - Maintaining eye contact;  - Drawing to explain;  - Explaining while using plain language;  - Being approachable through phone calls. |
| **Social and cultural determinants** | Relying on family members.  Alternative source of information (like Google). | Participation of family members in the decision making. |
| **Service-related determinants** | Nominal fees.  Tight examination duration. |  |
